# Activation-induced deoxycytidine deaminase (AID) co-transcriptional scanning at single-molecule resolution

**DOI:** 10.1038/ncomms10209

**Published:** 2015-12-18

**Authors:** Gayan Senavirathne, Jeffrey G. Bertram, Malgorzata Jaszczur, Kathy R. Chaurasiya, Phuong Pham, Chi H. Mak, Myron F. Goodman, David Rueda

**Affiliations:** 1 Department of Chemistry, Wayne State University, 5101 Cass Avenue, Detroit, Michigan 48202, USA; 2 Department of Biological Sciences, University of Southern California, Los Angeles, California 90089, USA; 3 Department of Medicine, Section of Virology, Imperial College London, Du Cane Road, London W12 0NN, UK; 4 Single Molecule Imaging Group, MRC Clinical Sciences Center, Imperial College London, Du Cane Road, London W12 0NN, UK; 5 Department of Chemistry, University of Southern California, Los Angeles, California 90089, USA; 6 Center for Applied Mathematical Science, University of Southern California, Los Angeles, California 90089, USA

## Abstract

Activation-induced deoxycytidine deaminase (AID) generates antibody diversity in B cells by initiating somatic hypermutation (SHM) and class-switch recombination (CSR) during transcription of immunoglobulin variable (IgV) and switch region (IgS) DNA. Using single-molecule FRET, we show that AID binds to transcribed dsDNA and translocates unidirectionally in concert with RNA polymerase (RNAP) on moving transcription bubbles, while increasing the fraction of stalled bubbles. AID scans randomly when constrained in an 8 nt model bubble. When unconstrained on single-stranded (ss) DNA, AID moves in random bidirectional short slides/hops over the entire molecule while remaining bound for ∼5 min. Our analysis distinguishes dynamic scanning from static ssDNA creasing. That AID alone can track along with RNAP during transcription and scan within stalled transcription bubbles suggests a mechanism by which AID can initiate SHM and CSR when properly regulated, yet when unregulated can access non-Ig genes and cause cancer.

## Abstract (web summary)

Activation-induced deoxycytidine deaminase (AID) induces somatic hypermutation and class-switch recombination during transcription of immunoglobulin genes. Here the authors use single-molecule FRET to show that AID translocates together with RNA polymerase and scans within stalled transcription bubbles.

## Graphical abstract

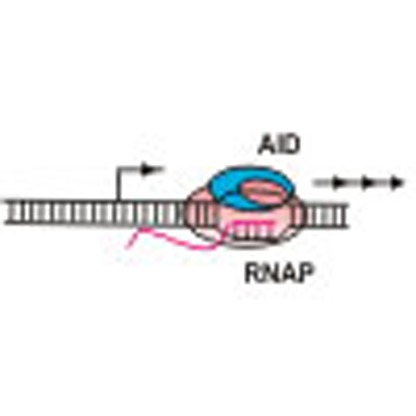

Expressed in B cells, activation-induced deoxycytidine deaminase (AID) plays an essential role in generating antibody diversity^1^,^2^. AID deaminates dC→dU on single-stranded (ss) DNA trinucleotide motifs in a sequence-dependent manner^3^. In the cell, AID acts during transcription of Ig variable (IgV) and switch (IgS) regions to initiate somatic hypermutation (SHM) and potentiate strand breaks required for class-switch recombination (CSR)^4^,^5^,^6^,^7^. AID favours the deamination of WRC motifs (W=A/T, R=A/G)^3^,^8^,^9^. However, a failure to regulate AID and related APOBEC-family proteins can generate spurious mutations implicated in a variety of cancers^10^,^11^,^12^,^13^,^14^,^15^,^16^.

SHM and CSR entail tightly regulated, complex biochemical interactions involving the selective recruitment of AID to transcriptionally active IgV and IgS regions^4^,^5^,^6^,^7^,^17^. Recently it was shown that ssDNA exposed during transcription initiation promotes the recruitment of AID to actively transcribed genes^18^. Furthermore, components of the RNA polymerase II transcription apparatus can interact with AID^4^,^5^,^6^,^7^,^19^,^20^. Although the mechanisms used to transport AID to transcribed IgV and IgS regions remain unresolved, transcriptional factors such as Spt4/5 may help recruit AID to stalled transcription bubbles^21^. Biochemical data showing that AID is catalytically inefficient, even when encountering favoured WRC motifs^3^,^8^,^9^, suggests the likelihood that deamination may be favoured in a stalled transcription bubble over a rapidly moving bubble^18^.

Single-molecule fluorescence resonance energy transfer (smFRET) can be used to observe the motion of individual molecules of AID and RNA polymerase (RNAP) while bound to DNA^22^,^23^. Here, we use smFRET to visualize the direct participation of AID in a T7 RNAP model transcription system in real time. In bulk solution studies with T7 RNAP, AID has been shown to perform transcription-dependent deamination favouring WRC motifs^3^,^24^,^25^. In this paper, we address how AID moves on DNA in the absence of auxiliary factors, either while subject to transcriptional constraints, or when scanning unconstrained on ssDNA.

We explore the time-dependent FRET interactions between AID and double-stranded (ds) DNA during transcription by directly tracking the location of AID along moving and stalled transcription bubbles. Initially, AID and RNAP are observed to co-localize on bubbles formed at the T7 promoter. AID then advances unidirectionally along the dsDNA in concert with moving transcription bubbles, while increasing the ratio of stalled-to-moving bubbles. We compare the scanning dynamics of AID confined in an 8-nt bubble with its unconstrained motion on single-stranded (ss) DNA. The time-dependent FRET interactions between AID and ssDNA show AID scanning in random short slides or hops over the entire ssDNA (72 nt), while remaining bound for ∼5 min.

## Results

### Visualizing RNAP during transcription

**Figure 1:**
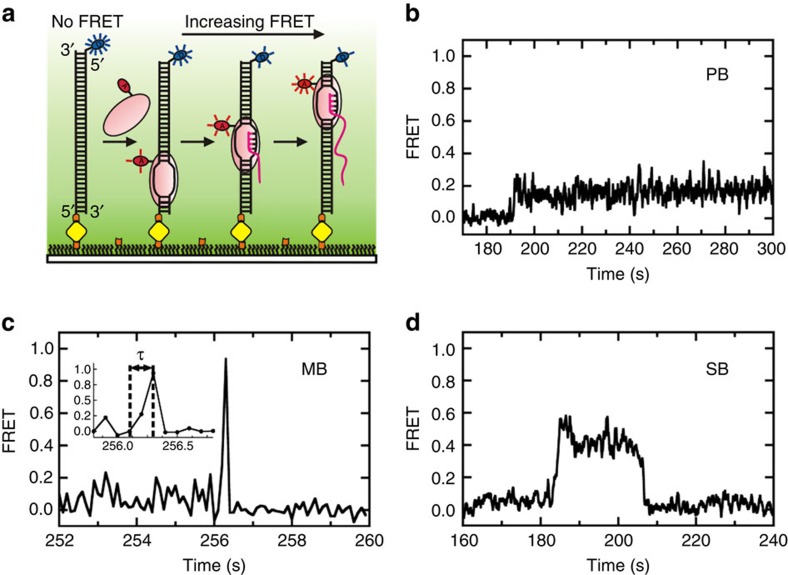
Single molecule FRET visualization of RNAP transcription. (**a**) Single-molecule FRET setup: surface-immobilized Cy3-labelled dsDNA (FRET donor in blue) with an RNAP promoter near the surface is used for transcription by Cy5-RNAP (FRET acceptor in red). In the presence of NTPs, three classes of events are observed for Cy5-RNAP: (**b**) promoter binders (PB), which are characterized as long static binding events at low FRET (∼0.2), (**c**) moving bubbles (MB) with a rapid increase to high FRET (≥0.5) followed by a sharp decrease to 0 FRET (inset shows the average peak rise time *τ*) and (**d**) stalled bubbles (SB) that rapidly increase to 0.2–0.5 FRET and remain there, before a sharp drop back to 0 FRET. The total number of events observed ranges from 78 to 87, as reported in Supplementary Table 2. The time resolution of FRET trajectories shown here is 100 ms, and these experiments were also conducted at 15–30 ms time resolution (Supplementary Figs 4–9).

First, we directly visualize RNAP behaviour in the absence of AID by smFRET. Several single-molecule studies have reported the transcriptional dynamics of T7 RNAP^26^,^27^,^28^,^29^,^30^. Here, we use Cy5-labelled RNAP to transcribe a Cy3-labelled 66-bp dsDNA construct attached to the surface of a microscope slide with biotin-streptavidin (Fig. 1a, Supplementary Table 1). Transcription initiates at the T7 promoter, near the slide surface (low FRET) and elongates towards the Cy3-labelled end of DNA (increasing FRET, Fig. 1a). In the absence of nucleoside triphosphate (NTP) substrates, all the observed events (Supplementary Table 2) reveal polymerase binding at the promoter site (∼0.2 FRET, Supplementary Fig. 1a). These events are not observed on a dsDNA construct lacking the promoter (Supplementary Fig. 1e).

We have moved the promoter to four different positions along the dsDNA (9, 19, 29 and 39 bp away from the Cy3-labelled end of the DNA) with a bound Cy5-labelled RNAP and in the absence of NTPs. We observed FRET increases that follow Förster's equation with an apparent *R*_0_=27±1 bp (Supplementary Fig. 1f). This apparent *R*_0_ value is larger than expected, which could be accounted for by the size of T7 RNAP, whose footprint is about 28 bp^31^, its orientation, the position of Cy5 and the length of its tether to RNAP (Supplementary Fig. 2), and distortions of the DNA structure (Supplementary Fig. 3). We note that if the DNA was an ideal B-form helix, this distance would correspond to ∼90 Å, or ∼3 nm longer than previously determined *R*_0_ values (∼18 bp) for Cy3–Cy5 (ref. 32).

In the presence of the four NTP substrates, we identify three classes of trajectories. The first class reveals RNAP binding at the promoter site (∼0.2 FRET, Fig. 1b, 31±8%) indicating promoter binders (PB) that fail to initiate transcription. The second class shows RNAP moving from the initial binding site (low FRET) towards the free dsDNA end (≥0.5 FRET), before running off the DNA (sharp drop to zero FRET, Fig. 1c, inset). The average peak rise time *τ* is 240±30 ms at 1 μM NTP (Supplementary Fig. 4a), and decreases with increasing NTP concentration (1–50 μM, Supplementary Figs 4a and 5), as expected for an RNAP transcription-related event. We assign these to moving bubbles (MB) that can potentially span the 39 nt region between the promoter and the distal dsDNA end. At 50 μM NTP, the average MB peak rise time is 129±15 ms, approaching our effective time resolution, and therefore observed event times may represent an upper limit. Representative donor and acceptor fluorescence intensities and corresponding FRET trajectories for each class are shown in Supplementary Fig. 6, and additional FRET traces for each class are shown in Supplementary Fig. 7. Labelling the RNAP with Cy3 and the DNA with Cy5 results in similar trajectories (Supplementary Fig. 8). We also observe trajectories in which the polymerase first binds the promoter at ∼0.2 FRET for up to ∼2 s, followed by translocation towards the distal end (high FRET) and then dissociation (Supplementary Figs 8d and 9).

A population analysis reveals that the fraction of MB trajectories increases from 11±4% to 54±11% with increasing NTP concentration (Supplementary Fig. 4b). The third class shows a rapid increase to intermediate FRET values (∼0.4 to 0.5) but remains there, until RNAP eventually unbinds or Cy5 photobleaches, consistent with stalled transcription bubbles (SB, Fig. 1d, 6±4% at 50 μM NTPs). Stalled transcription bubbles and PB can be readily distinguished because PB events remain bound to the DNA for very long periods, often longer than the experimental time window (Fig. 1b and Supplementary Fig. 6a), whereas SB events dissociate in an average time of 20±9 s (Fig. 1d and Supplementary Figs 6c and 8c). These assignments are consistent with previously reported transcription events^28^,^30^.

The template strand begins with three initial Cs and contains a single G near the centre (Supplementary Table 1). In the absence of GTP, no MB events are observed and 97±10% of the events are PB, as expected (Supplementary Fig. 4c). In the absence of CTP, the trajectories show PB (80±10%) and SB (20±4%, Supplementary Fig. 4c), consistent with transcription stalling at the single G. The low number of SB events observed may result from the rapid dissociation of RNAP in the absence of CTP. The ability to generate a discrete transcriptional stopping point midway between the promoter and free end (0.4 FRET) further supports our assignments of PB, MB and SB.

### AID translocates with RNAP during transcription

**Figure 2:**
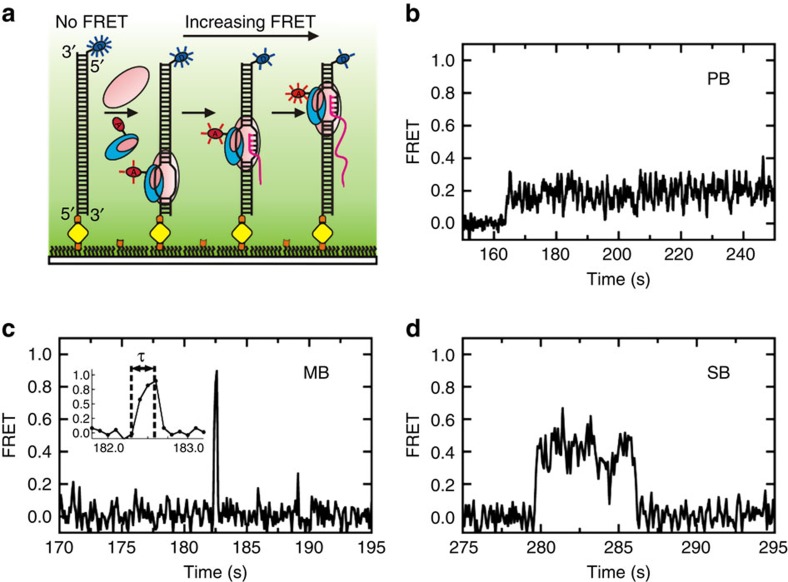
AID co-transcriptionally scans dsDNA. (**a**) Single-molecule FRET setup for visualizing co-transcriptional AID scanning. A Cy3-labelled dsDNA (FRET donor in blue) with an RNAP promoter near the surface is surface-immobilized and RNAP transcribes the DNA. AID is labelled with Cy5 (FRET acceptor in red) to monitor its dynamics during transcription. (**b**–**d**) Representative FRET trajectories (*N*=55) showing (**b**) AID binding to the promoter region in the presence of NTPs, (**c**) AID moving along with RNAP (MB) and (**d**) AID scanning in a stalled transcription bubble (SB). The time resolution of FRET trajectories shown here is 100 ms, and these experiments were also conducted at 15–30 ms time resolution (Supplementary Figs 4 and 5).

AID deaminates dC→dU on ssDNA and transcribed dsDNA substrates (Supplementary Fig. 10)^3^,^8^,^24^,^25^,^33^,^34^. To visualize transcription-dependent AID scanning on dsDNA by smFRET, we used Cy5-AID and unlabelled RNAP (Fig. 2a). Cy5-AID retains the same deamination properties as the unlabelled enzyme (Supplementary Fig. 11). The experiment with Cy5-AID and unlabelled RNAP is essentially the same as that for Cy5-RNAP and unlabelled AID: transcription initiates at the tethered promoter, and elongation proceeds towards the Cy3-labelled DNA end (Fig. 2a).

For AID and RNAP, we observe the same three classes of trajectories (PB, MB and SB) that occurred for RNAP in the absence of AID. RNAP-dependent binding of Cy5-AID to the promoter occurs in a low FRET state (Fig. 2b, PB, FRET ∼0.2, 35±9%). Although the PB average FRET values are similar for the Cy5-AID and Cy5-RNAP experiments, centred near 0.2 FRET, their FRET distributions differ, indicating that AID and RNAP are not static relative to each other on the initiation bubble. AID has a distinct FRET signature. As the binding of AID to dsDNA requires the presence of RNAP, the observed FRET of Cy5-labelled AID to the Cy3-labelled distal end of the dsDNA is also related to Cy5-labelled RNAP. Yet the Cy5-RNAP distribution is broader than that for Cy5-AID, indicating that the Cy5-RNAP distribution is more heterogeneous. This could be caused by dynamic motion of RNAP on the template DNA, while AID motion might be somewhat more constrained when bound to the non-template strand.

Translocation of AID with a moving transcription bubble is detected by a rapid increase of FRET to ∼0.9 followed by a sharp loss of FRET (Fig. 2c and inset, MB, 33±9%). In the presence of Cy5-AID, the average MB peak rise time increases to 182±25 ms (Supplementary Fig. 5d), indicating that either AID moves slower than the polymerase or that AID binding to the transcription bubble slows the rate of RNAP elongation. In the presence of AID, the fraction of SB events increases four-fold (6±4% to 25±8%, Supplementary Fig. 4d), suggesting the possibility that AID induces stalling, thus providing additional time for scanning and deamination.

Stalled bubbles are identified by a rapid increase to intermediate FRET values, ∼0.4 to 0.6, that remain roughly static for about 6 s (Fig. 2d, SB, 33±9%). Cy5-AID does not bind to dsDNA in the absence of RNAP (Supplementary Fig. 12a). When Cy5-AID and RNAP are present but NTPs are absent, we observe long-lived static binding of AID at the promoter (∼0.2 FRET, PB, Supplementary Fig. 12b). These events are not observed on dsDNA lacking a T7 promoter (Supplementary Fig. 12c).

Representative donor and acceptor fluorescence signals and corresponding FRET trajectories for each event are shown in Supplementary Fig. 13. Moving bubbles have the potential to span the 39 nt region between the promoter and the distal dsDNA end, indicating AID motion away from the tethered promoter towards the free end, followed by enzyme dissociation. The presence of Cy5-AID increases the MB peak time by ∼1.4-fold, indicating that AID may reduce the rate of RNAP elongation (Supplementary Fig. 4a), while concomitantly increasing the ratio of stalled-to-moving bubbles (Supplementary Fig. 4d). This effect is more pronounced with Cy5-AID because all the measured MB events contain AID, whereas in the presence of Cy5-RNAP and unlabelled AID, not all observed MB include AID (Supplementary Fig. 4a).

### AID and RNAP co-localize at transcription initiation sites

**Figure 3:**
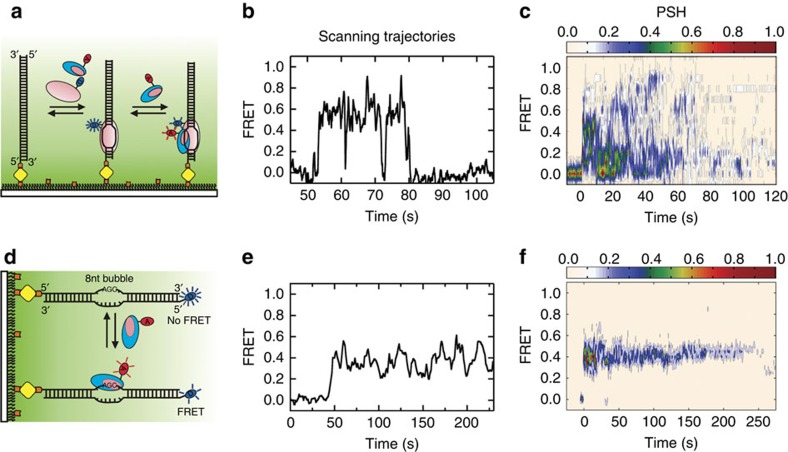
AID co-localizes with RNAP in transcription initiation bubbles and scans static ssDNA bubbles. (**a**) Cy3-RNAP and Cy5-AID incubated with surface-immobilized unlabelled dsDNA show co-localization of both enzymes within the transcription initiation bubble. (**b**) The resulting FRET trajectories exhibit fluctuations indicating AID dynamically co-localizes with RNAP, moving on the DNA relative to the polymerase (*N*=16). (**c**) Post-synchronization histogram (PSH) showing AID co-localizes with RNAP at the initiation bubble. PSHs are time-binned trajectories synchronized at the initial binding event. Intensity bar represents the frequency scale of particular FRET states (beige=low, red=high). (**d**) Single-molecule FRET setup for detection of AID scanning in a static transcription bubble. A Cy3-labelled dsDNA with an 8 nt bubble in the middle was immobilized on the surface, and Cy5-labelled AID was used to bind/scan in the bubble. (**e**) A representative smFRET trajectory (*N*=18). (**f**) PSH showing restricted AID scanning within the bubble. The time resolution here is 100 ms.

To show that AID and RNAP can simultaneously bind within a transcription initiation bubble, we introduced Cy3-RNAP and Cy5-AID on unlabelled dsDNA (Fig. 3a). A representative single-molecule trajectory (Fig. 3b) exhibits a sizable range of FRET values, indicating that the two enzymes co-localize on the DNA. A post-synchronization histogram (PSH) of the ensemble of trajectories (Fig. 3c) aligned at the initial binding event^35^ shows density ranging from 0.1 to 0.9 FRET, confirming that AID is likely not interacting directly with RNAP. The FRET fluctuations indicate relative motion between the two enzymes, possibly suggesting that AID scans the transcription initiation bubble while RNAP is stalled, consistent with Fig. 2d. No direct interactions between AID and T7 RNAP were detected, as determined by the absence of rotational anisotropy changes of fluorescein-labelled RNAP with increasing AID concentrations (Supplementary Fig. 14).

### AID scans randomly while constrained in an 8-nt DNA bubble

To test whether AID is able to scan within a small static DNA bubble, we introduced an 8-nt mismatch into the dsDNA construct (Fig. 3d). The single-molecule trajectories show that AID binds the DNA for up to ∼250 s and localizes to a narrow region within the bubble (0.2–0.6 FRET, Fig. 3e). To eliminate data blurring from asynchronous binding, the trajectories were post-synchronized by alignment at the initial binding event^23^,^35^. The PSH (Fig. 3f) shows an initial binding at ∼0.4 FRET, followed by fluctuations between 0.3 and 0.55 FRET, confirming that AID moves randomly and bidirectionally over a narrow region of the DNA. The ability of AID to scan a static bubble for up to 250 s would in principle allow sufficient time for AID to catalyse dC deaminations in spite of its exceedingly low efficiency (∼3% in favoured WRC motifs)^8^,^9^. The smFRET data are consistent with previous biochemical data showing that AID deaminates dC within static bubbles^36^,^37^,^38^.

### AID moves in short random slides/hops on ssDNA

**Figure 4:**
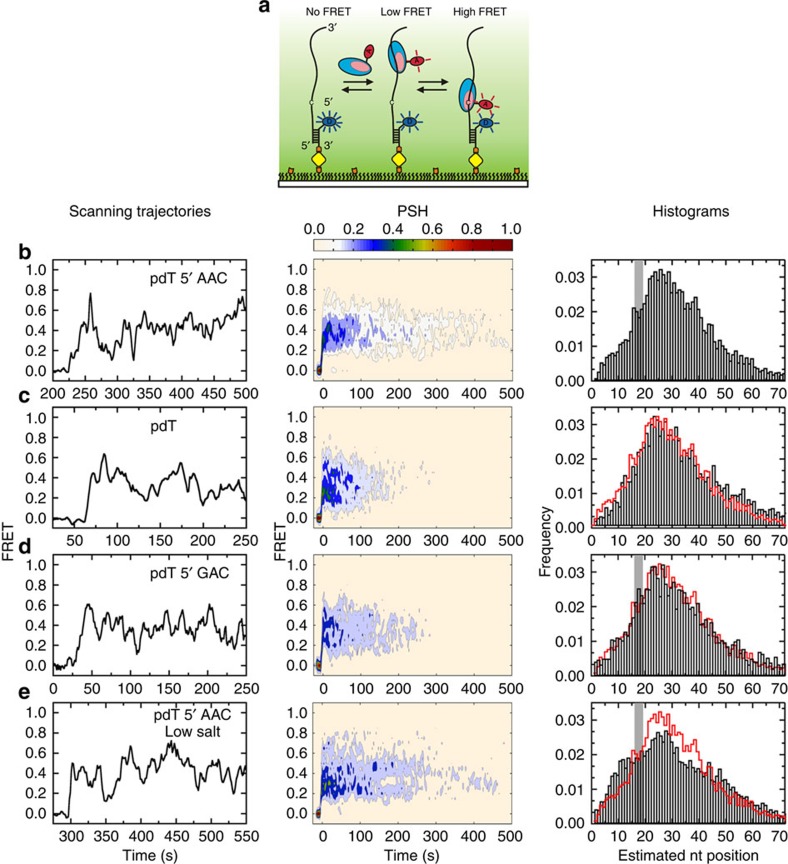
AID scans ssDNA randomly and bidirectionally. (**a**) Single-molecule FRET setup for analysing scanning of AID on ssDNA. A 92-nt ssDNA construct is annealed to a Cy3-labelled surface-immobilized anchor DNA. Binding and scanning of Cy5-AID results in FRET changes. (**b**–**e**) Representative smFRET time trajectories (panel 1, *N*>100), PSH (panel 2) and estimated nucleotide position histograms (panel 3, target motif location highlighted in grey). (**b**) Representative smFRET scanning trajectory on the 5′-AAC hot substrate shows an increase to ∼0.2 FRET upon initial binding of AID, followed by fluctuations centred around ∼0.4 FRET (PSH), for as long as ∼400 s. A histogram shows the estimated nucleotide positions visited during AID scanning events. (**c**) Eliminating the AAC hot motif substantially reduces the tail of the PSH. The histogram suggests AID has an apparent preference for the 5′ end, with a peak around nucleotide position 25. It is possible that the high sensitivity of FRET between nt positions 10 and 60 (Supplementary Fig. 18b) skews the correlation between FRET and nt position. AID also creases the substrate at long distances (Supplementary Fig. 18b–c, grey squares), and may become trapped at the position of ssDNA bend formation. However, the sequence position histograms clearly show that AID scans the entire ssDNA substrate. (**d**) Substituting the AAC hot motif with a GAC non-hot motif substantially reduces PSH tail near ∼0.4 FRET, but does not change the sequence position histograms (panels 2 and 3). (**e**) Decreasing the salt concentration does not significantly alter the tail of the PSH, but broadens the sequence position histogram (panels 2 and 3). The time resolution here is 1 s.

As AID acts only on ssDNA, we sought to characterize the unconstrained scanning of Cy5-AID on ssDNA (Fig. 4a). Scanning dynamics were compared using pdT substrates (72-mer) containing an AAC hot motif (Fig. 4b), no motif (pdT, Fig. 4c), GAC non-hot motif (Fig. 4d), AAC hot motif at low salt (Fig. 4e) and AAU product motif (Supplementary Fig. 16). The smFRET trajectories (Fig. 4 and Supplementary Fig. 15) show AID binding as an abrupt increase from zero FRET (Fig. 4b, ∼225 s), and scanning motion as changes in FRET efficiency (Fig. 4b, ∼225–480 s). Motion towards the 5′ and 3′ ends are observed as increases and decreases in FRET, respectively. Over 80% of AID molecules remain bound to ssDNA for 25 s–10 min, with an average residence time of 270±30 s (∼4.5 min, Supplementary Table 3). These long binding trajectories display rapid fluctuations with a FRET efficiency ranging from 0.2 to 0.8, indicating that AID scans the entire ssDNA (72 nt), moving in processive slides/hops between the tethered 5′ end (∼0.8 FRET) and free 3′ end (∼0.2 FRET) with equal probability, indicating random and bidirectional scanning (Supplementary Fig. 16). This remarkably long binding duration is illustrated in Supplementary Fig. 15 and the Supplementary Movie. A minority population of short binders (<15%) is also present with residence times <25 s.

Long binding trajectories were post-synchronized by alignment at the initial binding event^35^. The PSH reports on the memory of AID's motion on the ssDNA in terms of FRET values (positions on the DNA). For the AAC hot motif, the PSH shows an initial increase to ∼0.2 FRET (Fig. 4b), consistent with the initial binding observed in the scanning trajectories. Then, the FRET increases to ∼0.4, followed by persistent oscillations between 0.2 and 0.8 (Δ≈0.6), confirming that AID moves randomly and bidirectionally over the entire ssDNA length.

The FRET trajectories reveal details of how AID scans its substrate, but as individual FRET states cannot be identified, we correlate FRET values to estimated substrate position using an empirical calibration with doubly labelled ssDNA (Supplementary Figs 17 and 18). This correlation allows us to map FRET to sequence position in an average sense, which we use to build histograms that reveal how often AID visits a particular nt position on the DNA (Fig. 4, histograms). The histogram confirms that AID scans the entire length of the DNA.

The PSH plots provide a scanning timescale (which is not present in the histograms), indicating a longer time persistence of FRET signal in the rough vicinity of a single hot motif (AAC, Fig. 4b) compared with either no motif (pdT, Fig. 4b), or to a non-hot motif (GAC, Fig. 4d). This distinction appears most pronounced when comparing the long tail for AAC (∼300–400 s) with pdT (∼100–200 s). However, the histograms depicting the spatial scanning distributions are all similar and do not distinguish between motifs. However, when dC is replaced by dU, the FRET oscillations in the PSH move toward low FRET values (the 3′-free DNA end), persisting for ∼300 s, and the corresponding histogram shifts toward the right (Supplementary Fig. 19). The average residence time for all constructs with and without C remain comparable (∼4 min, Supplementary Table 3), consistent with the lack of sequence specificity in AID-ssDNA binding affinity^39^.

**Figure 5:**
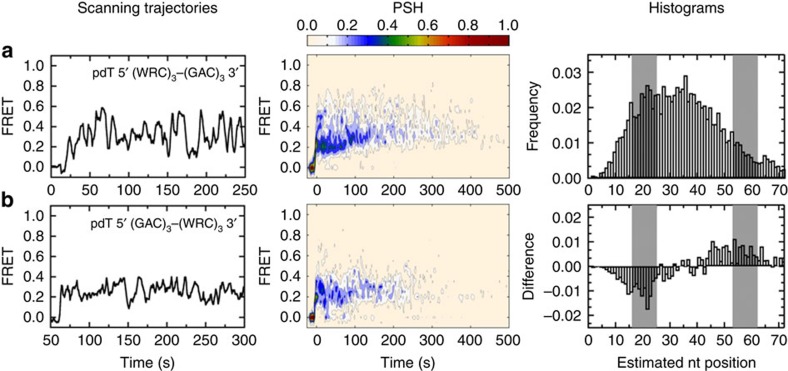
AID scanning in the presence of multiple deamination targets. Representative smFRET time trajectories (panel 1, *N*>100), PSH (panel 2) and estimated nucleotide position histograms (panel 3, motifs highlighted in grey). Experimental set up is the same as that shown in Fig. 4a, and all the long binding transitions used for the analyses are summarized in Supplementary Table 2. (**a**) Placing multiple motifs on the same ssDNA constructs further characterizes the scanning of AID. On the pdT 5′ (WRC)_3_ 3′ (GAC)_3_ construct, AID scans the entire DNA, as indicated by the wide FRET range (0.15–0.7) and the pronounced PSH ∼400 s long tail. The estimated nucleotide position histogram is also broader than that for the single 5′ hot motif. (**b**) Relocating the tandem hot motifs from the 5′ end to the 3′ end of the ssDNA moves the FRET probability in the same direction, as indicated by the lower FRET range (0.1–0.4), the shorter PSH tail (∼300 s, panel 2), and the probability shift in nucleotide position towards the 3′ end (difference histogram, panel 3). The time resolution here is 1 s.

In an attempt to characterize the behaviour of AID in the presence of multiple deamination motifs, we investigated constructs containing three hot motifs located either towards the 5′ or 3′ end (Fig. 5a,b, respectively; note that we have used 5′-AACAGCAAC for the hot motifs instead of 5′-AACAACAAC to avoid secondary structure formation). The PSH tails for the multiple hot motifs extended out to ∼300–400 s (Fig. 5a,b, middle panels), which is similar to that for the single hot motif (Fig. 4b). The FRET histograms are wider than those for the single motifs constructs. A difference histogram between the two constructs (Fig. 5b, right panel) shows that moving the hot motifs towards the 3′ end results in a probability decrease at high FRET (around the location of the 5′ end non-hot motifs) and a probability increase towards low FRET (around the location of the 3′ end hot motifs). The PSH also shows a shift towards low FRET values. These differences may account for sequence-dependent deamination differences, whose molecular mechanism remains unclear^40^. Interestingly, the average residence for all constructs is comparable (∼4 min, Supplementary Table 3), consistent with the lack of sequence specificity in AID-ssDNA binding affinity^39^.

### AID motion is similar at high- and low-salt concentrations

There are no major differences in FRET trajectories, PSH, estimated position histograms and binding lifetimes when AID scans ssDNA in ‘high' ionic strength (60 mM Na^+^, 5 mM Mg^2+^, Fig. 4b) compared with ‘low' ionic strength (30 mM Na^+^, no Mg^2+^, Fig. 4e). The low-salt position histogram is slightly broader than that in high salt, indicating a minor increase in scanning (Fig. 4e, histograms). The absence of a significant scanning dependence on salt for AID is strikingly different from Apo3G, which shows a marked reduction in scanning lengths at low salt^23^. As hopping might be more readily interrupted in the presence of salt than sliding^41^, this result suggests that Apo3G favours hopping over sliding, whereas AID movement along the ssDNA might tend to favour sliding. Movement predominantly by sliding is consistent with the presence of similar tightly clustered dC deamination patterns observed at both low and high ionic strength^8^ relative to Apo3G^23^.

### Scanning motion of AID involves excursions of many sizes

**Figure 6:**
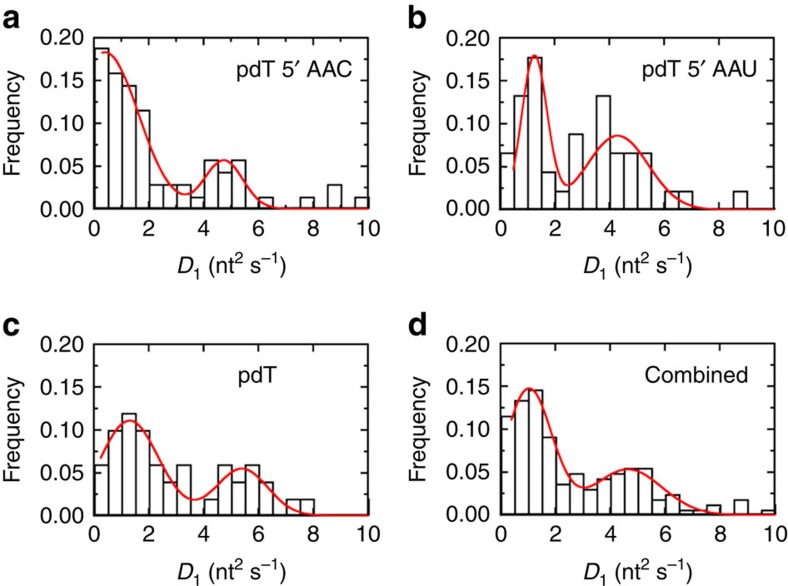
Diffusion coefficient histograms from MSD analysis. Distribution of one-dimensional diffusion coefficients obtained from mean square displacement analysis (see Supplementary Fig. 20) of (**a**) 5′ AAC, (**b**) 5′ AAU, (**c**) pdT and (**d**) all the three constructs combined. The distribution of diffusion coefficients has two main populations. The primary population (centred at *D*_1_=1 nt^2^ s^−1^) corresponds to trajectories characterized by localized dynamics over a narrow range of nucleotide positions (Supplementary Fig. 20a), while the secondary population (*D*_1_=5 nt^2^ s^−1^) corresponds to trajectories characterized by long excursions traversing the entire length of substrate (Supplementary Fig. 20b). The time resolution here is 1 s.

On a local scale, AID scans randomly and bidirectionally. However, understanding the large length-scale ‘diffusive' motion of AID on ssDNA is significantly more challenging because the limited sequence length of the substrate interrupts these diffusive motions. To characterize the diffusive behaviour of AID, we estimate the mean square displacement (MSD) from FRET trajectories of the hot motif (*N*=72), the product motif (Supplementary Fig. 20, *N*=52) and pdT (*N*=54; ref. 41). First, we estimate the nucleotide position of AID using the distance calibration (Supplementary Fig. 17a–c). Next, we calculate the MSD for each trajectory as a function of time (Supplementary Fig. 20d), the slope of which yields individual diffusion coefficients. Finally, we build a histogram with the distribution of diffusion coefficients for each construct (Fig. 6a–c). As the distributions do not vary substantially across constructs, we combine them to ascertain the nature of the large length-scale motions of AID (Fig. 6d). The combined distribution exhibits a broad range of diffusion coefficients centred on two populations. The major population (*D*_1_ ∼1 nt^2^ s^−1^) consists of trajectories characterized by motions covering a narrow range of nucleotide positions (Supplementary Fig. 20a), while the minor population (*D*_1_ ∼5 nt^2^ s^−1^) comprises trajectories characterized by larger excursions traversing the entire length of substrate (Supplementary Fig. 20b). The broad distribution is qualitatively consistent with a previous mathematical modelling estimate of AID scanning transition rates^9^. To produce a distribution with higher statistical confidence, we have pooled data from Fig. 6a–c and from other substrate constructs to produce the combined histogram in Fig. 6d.

### Resolving AID scanning from ssDNA creasing

An important question is whether the observed FRET changes are *bona fide* ssDNA scanning or protein-induced creasing of the intervening DNA. We use the term ‘creasing' instead of ‘bending' to denote AID-induced conformational changes in ssDNA, and distinguish that from bending of dsDNA, which involves persistence lengths that are absent in ssDNA. To distinguish these two scenarios, we moved the donor to the free 3′ end of ssDNA (Supplementary Fig. 21). In this configuration, constant high FRET is expected if the protein binds near the 3′ end at a fixed location while creasing the intervening DNA. The observed trajectories clearly show long binding events that display oscillations ranging 0.2–0.8 FRET, consistent with AID scanning the entire ssDNA. Dynamics of the ssDNA tail are expected to occur on a millisecond timescale^42^, which cannot account for the seconds-to-minutes timescale associated with AID scanning.

In the presence of AID, the FRET calibration distributions broaden, indicating AID binding and some protein-induced creasing. Broadening becomes more pronounced for the constructs labelled near the 3′ end (dT60 and 72), as expected. However, the resulting average distance for dT7-45 remains essentially unchanged (Supplementary Fig. 18), showing that in this region protein-induced creasing only minimally affects our ability to track protein scanning. Comparing the Cy5-AID FRET histogram with the distributions of dT7-60 (Supplementary Fig. 22) shows that the Cy5-AID histogram spans a wider FRET range, indicating that the protein scans at least 60 nt of the DNA length. The FRET changes observed in Fig. 4 must therefore reflect scanning. Creasing only becomes significant between 60 and 72 nt, near the 3′ end of the DNA. AID-induced creasing of long ssDNA may help the enzyme traverse sizable distances in primary structure.

## Discussion

Mouse and cultured cell models have been used to address how AID targets transcribed IgV- and IgS-DNA^19^,^20^. Proteins that co-immunoprecipate with AID have been proposed to play a role in targeting^43^,^44^,^45^,^46^,^47^,^48^,^49^,^50^. For example, the transcription factor Spt5, which is believed to stall transcription^21^, may allow sufficient time for the low-efficiency AID^8^,^9^ to deaminate dC on the transiently exposed single-stranded region of a transcription bubble. However, a biochemical basis for targeting is not known. We, and others, have reported that AID catalyses dC→dU during transcription by T7 RNAP, primarily on the non-transcribed strand^3^,^8^,^25^,^34^,^51^. Here, we have used smFRET to investigate the constrained dynamics of AID during transcription of dsDNA and unconstrained motion when scanning ssDNA.

When initiating SHM and CSR, AID gains access to IgV and IgS regions undergoing transcription in B cells. Transcribed S regions are composed of large R-loops several hundred nts long that can provide ample tracts of ssDNA on which AID can move more or less freely^52^,^53^. However, when acting on V regions, AID is constrained to act locally within small transcription bubbles (∼8 to 12 nt). The bubbles can either be moving rapidly and unidirectionally away from the promoter or be transiently stalled. To determine the location and motion of AID relative to active RNAP, we have visualized transcription events with single-molecule resolution in real time in the presence and absence of AID.

In the absence of AID, three classes of trajectories are observed: PB, MB and SB (Fig. 1). These same three classes are observed in the presence of AID (Fig. 2), but with two key differences: MB timescales increase and the fraction of SB increases, indicating that AID may slow translocation of RNAP and possibly induce stalling (Supplementary Fig. 4).

**Figure 7:**
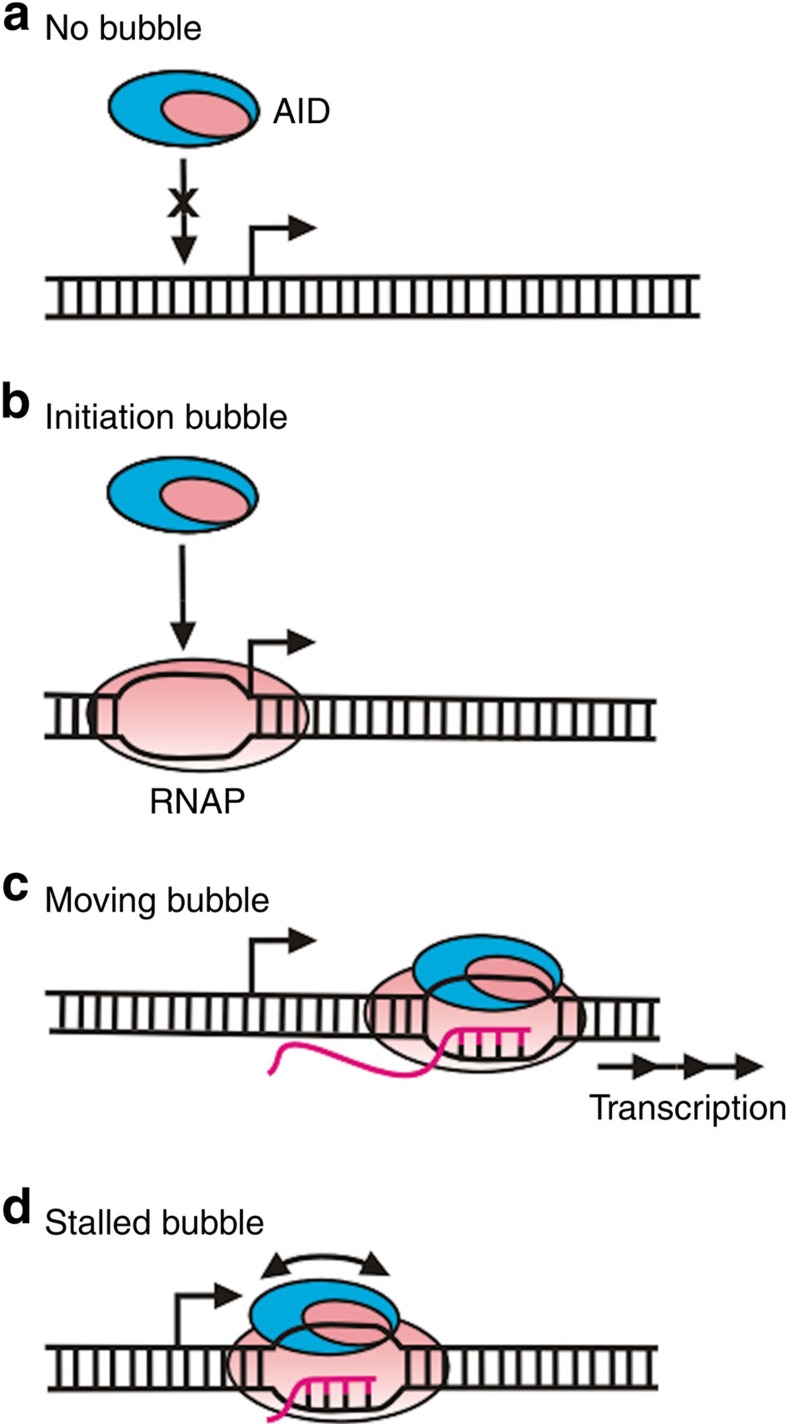
Model for co-transcriptional scanning of AID. (**a**) AID does not bind to dsDNA in the absence of T7 RNAP. (**b**) Following the formation of a transcription initiation bubble at the T7 promoter by RNAP, AID binds to the bubble with RNAP. (**c**) AID translocates unidirectionally along with RNAP on a moving bubble. (**d**) AID scans bidirectionally in short slides/hops within a stalled transcription bubble (indicated by the double-headed arrow). The presence of AID retards the RNAP translocation rate and increases the ratio of stalled to moving bubbles.

The PB, MB and SB trajectories observed with Cy5-RNAP are similar to traces with Cy5-AID and RNAP (compare Figs 1 and 2), suggesting that AID and RNAP motion is closely coupled. By tracking the motion of Cy5-AID during transcription, we determined that AID: (1) binds at the promoter in the presence and absence of NTP substrates, (2) translocates unidirectionally away from the promoter along with RNAP during rapid transcription, (3) scans the stalled transcription bubble and (4) co-localizes with RNAP during transcription initiation but does not interact directly. Therefore, the non-template strand likely mediates the interaction between AID and RNAP in static and moving transcription bubbles. A model sketch of the co-localized binding of AID and RNAP at the T7 promoter and on moving and stalled bubbles is shown in Fig. 7.

AID binds DNA essentially randomly and scans ssDNA in random bidirectional short sliding or hopping movements (Figs 4 and 5). AID deamination activity is increased by ∼10-fold at AAC motifs compared with GAC^8^, suggesting perhaps that higher enzymatic activities of AID acting on hot motifs could result from increased contact time between AID with WRC motifs. We tried to detect an increase in contact time by investigating whether FRET trajectories could be observed for extended periods of time at hot versus not-hot motif locations, and in relation to no motifs (pdT). This analysis, however, proved inconclusive. We nevertheless point out that AID deamination efficiencies are remarkably small, ∼1–7% for WRC hot motifs^8^,^9^. Thus, when confronting a hot motif, catalysis occurs <10% of the time, which leads us to speculate that the extended PSH tail for AAC compared with GAC (compare Fig. 4b, d) might occur from a transient retardation of AID motion tied to motif recognition and inefficient catalysis.

AID scans multiple times along the entire length of the DNA during a single binding event (processive scanning, Figs 4 and 5). The mean lifetime for AID bound to ssDNA is ∼4.5 min (Supplementary Table 3 and Supplementary Movie), in agreement with the residence time of AID on ssDNA determined from processive introduction of multiple deaminations on the same DNA substrate over 5 min (ref. 9). However, owing to the structural flexibility of ssDNA, AID can also crease the backbone (Supplementary Fig. 18). Therefore, it is essential to distinguish between FRET changes owing to AID motion (slides/hops) from those owing to AID-induced ssDNA creasing. AID scanning is identified unambiguously by FRET traces that are essentially indistinguishable when Cy3 is placed at either end of the DNA (Fig. 4b and Supplementary Fig. 21). When Cy5-AID and Cy3-DNA are <60 nt apart, the FRET changes arise predominantly from AID scanning. However, when Cy5-AID and Cy3-DNA are ≥60 nt apart, both scanning and creasing are observed (Supplementary Fig. 18c).

The high processivity of AID could well have important biological ramifications for the well-documented adventitious targeting of AID to non-Ig regions of genomic DNA. A number of genes rich in short clustered repeat sequences with AID hot motifs similar to V and S regions have been shown to be highly susceptible to AID activity^54^. For example, SNHG3, MALAT1, BCL7A and CUX1 all have AID-dependent mutations with frequencies comparable to SHM at IgV regions (2.2 × 10^−4^ to 6.1 × 10^−4^)^54^.

This study represents the first visualization of AID motion relative to actively transcribing RNAP and alone on ssDNA. When operating in the wrong place and/or the wrong time, AID and other APOBEC dC deaminases (Apo3A, B and G) have been implicated in different types of cancer^10^,^11^,^12^,^13^,^14^,^15^,^55^. That AID alone can track along with RNAP during transcription, and scan within a stalled transcription bubble, suggests that it might behave similarly in the cell (Fig. 7). Therefore, while it is clearly essential to identify proteins that target AID to its proper IgV- and S-target regions, it might be equally important to identify ‘anti-targeting' proteins designed to ensure that AID cannot gain access to transcribed non-IgV or IgS DNA, or tracts of ssDNA generated during aberrant recombination or repair of genomic DNA.

## Methods

### Cy5- and Cy3-labelled AID and T7 RNAP for smFRET

A carboxy (C)-terminal hexa-His-tagged GST-AID variant was expressed in *Sf*9-infected insect cells and purified as follows^3^,^36^. Infected *Sf*9 cells were suspended in lysis buffer containing 20 mM HEPES pH 7.5, 150 mM NaCl, 1 mM DTT, 1 mM EDTA, 10 mM NaF, 10 mM NaHPO_4_ and 10% glycerol. The cells were lysed by sonication, and the crude lysate was cleared by centrifugation at 26,000*g*. The supernatant containing GST-AID was incubated with glutathione sepharose resin for 1 h and then washed extensively with 50 mM HEPES pH 7.5, 1 M NaCl and 10% glycerol. The purified protein was site-specifically labelled with Cy5 fluorophores using a Cy5-OEG-TrisNTA conjugate^56^,^57^, which forms a stable complex with the hexa-histidine residues. Cy5-OEG-trisNTA was synthesized and loaded with nickel ion in-house. Cy5-OEG-trisNTA labelling was performed for 1 h, at 4 °C during protein purification in 50 mM HEPES (pH 7.5, 1 M NaCl). Excess label was removed by washing the protein while stably bound to GST resin. After the collection of labelled protein fractions, glycerol was added to a final concentration of 10% and Cy5-GST-AID was stored at −80 °C. Protein concentration was determined by Bradford Assay and bovine serum albumin (BSA) protein standardization. Labelling efficiency was determined by absorption at 280 and 650 nm. Cy5-labelled GST-AID retained the activity and deamination properties of unlabelled GST-AID (Supplementary Fig. 11).

T7 RNA polymerase containing hexa-histidine residues at the amino (N) terminus (His_6_-RNAP) was overproduced and purified from *Escherichia coli* as follows. BL21 (DE) cells harbouring pBH161 vector^58^ were grown at 37 °C until *D*_600_=0.6–0.7 and 1 mM isopropyl-β-D-thiogalactopyranoside was added to induce expression. The cells were collected 4 h after induction, resuspended in a lysis buffer containing 50 mM sodium phosphate pH 8.0, 500 mM NaCl, 1 mM dithiothreitol (DTT), 0.1% Triton-X, 5 mM imidazole, 1 mg ml^−1^ lysozyme and protease inhibitor cocktail (Roche) and lysed by sonication. The crude lysate, containing soluble His_6_-RNAP was collected after centrifugation at 17,000*g* for 30 min followed by loading onto a 5 ml Ni-NTA resin column (Qiagen). After extensively washing the column with the lysis buffer supplemented with 10 mM imidazole, the bound protein was eluted from the Ni-NTA resin using 20–250 mM imidazole gradient. Peak eluted fractions containing His_6_-RNAP were collected, dialysed in a storage buffer (20 mM HEPES pH 7.8, 100 mM NaCl, 1 mM DTT and 10% glycerol) and stored at −80 °C. His_6_-RNAP was labelled with Cy5-OEG-TrisNTA or Cy3-OEG-TrisNTA. Labelling was performed at 4 °C for 1.5 h in labelling buffer (50 mM HEPES pH 7.8, 500 mM NaCl). Excess label was removed using a gel-filtration column (Bio-gel P6, Bio-Rad), equilibrated with 20 mM HEPES pH 7.8, 100 mM NaCl and 10% glycerol.

### DNA oligonucleotides

All DNA oligonucleotides used for this study (Supplementary Table 1) were synthesized in-house using a 3400 DNA synthesizer (Applied Biosystems) or ordered from Operon. The DNA was purified using polyacrylamide gel electrophoresis^23^. Biotin modifications and some of the Cy-dye modifications were inserted into the oligomers whenever needed as phosphoramidites. In other instances, Cy3 or Cy5 were incorporated into the 5′ or 3′ termini of the DNA by reacting the NSH-ester form of the dye (GE Healthcare) with amino-modified C6-dT. For C6-dT labelling, ∼1 nmol of desalted DNA was dissolved in 44 μl of 100 mM sodium tetraborate buffer, pH 8.5 and mixed with ∼10 nmol of Cy-dye dissolved in 7 μl of dimethyl sulphoxide, vortexed several times, and incubated at room temperature overnight to obtain ≥80% labelled DNA. Finally, labelled and unlabelled DNA fractions were separated by reverse-phase high-performance liquid chromatography chromatography on an analytical C18-column. Purified oligonucleotides were stored at −20 °C in 10 mM Tris-HCl, pH 8.0.

### smFRET for co-transcriptional AID scanning

To minimize nonspecific binding, quartz microscope slides were passivated with methoxy-PEG-SVA (*M*_r_=5,000; Laysan Bio Inc.), doped with 8% biotin-PEG-SVA (*M*_r_=3,400; Laysan Bio Inc.) and further treated by incubating the reaction chamber with 0.2 mg ml^−1^ BSA (Sigma) in T50 buffer (50 mM Tris-HCl, pH 7.0, 50 mM NaCl) for ∼10 min. After washing out excess BSA with T50 buffer, streptavidin (1 mg ml^−1^ in T50 buffer) was incubated for ∼10 min and then washed out. DNA samples were prepared by annealing the two complementary ssDNA constructs (Supplementary Table 1) in annealing buffer (10 μl, 50 mM MOPS, pH 7.4, 5 mM Mg^2+^, 60 mM Na^+^ and 2 mM Trolox) by heating to 90 °C for 45 s and cooling to room temperature over 15 min, followed by serial dilutions to 25–50 pM of partial dsDNA for surface immobilization through biotin–neutravidin–biotin linkage. In these experiments, a Cy3-labelled dsDNA with a T7 promoter near the surface was immobilized and T7 RNAP polymerase (0.5–5 nM) was used to transcribe the DNA. A total 20 nM Cy5-labelled AID was used to bind and scan in the transcription bubbles at varying NTP concentration in a transcription-scanning buffer (40 mM Tris-HCl, pH 7.9, 5 mM Mg^2+^, 60 mM Na^+^, 0.2 mg ml^−1^ BSA, 2 mM Trolox to prevent photo-blinking of the dyes and oxygen scavenger system consisting 2.5 mM 3,4-dihydroxybenzoic acid (PCA-SIGMA), 250 nM protocatechuate dioxygenase (PCD-SIGMA) to minimize the photobleaching of the dyes). Data were acquired on a prism-based smTIRF microscope at 15 ms and 100 ms time resolution^59^. Movies were recorded from ∼10 different areas of a sample chamber for ∼5 min each. Apparent energy transfer efficiencies were obtained as FRET=*I*_A_/(*I*_D_+*I*_A_), where *I*_A_ is the acceptor intensity and *I*_D_ is the donor intensity. Although less than 10% of single-molecule trajectories exhibit RNAP-binding events, such yields are considered typical in single-molecule experiments. The resulting trajectories were divided in PB, MB or SB populations, as described in the text. An arbitrary 0.5 FRET cut-off value was chosen at the middle of the FRET range. Decreasing it does not notably change the population analysis, but increasing it will result in larger SB populations and lower MB populations. The PB population is not affected by this cut-off value.

### smFRET for Cy5-AID scanning transcription bubble dsDNA

A Cy3-labelled, 66-nt long, partially complementary dsDNA, with an 8-nt ‘transcription bubble' in the middle was obtained by annealing a 5′-biotin 70mer DNA to a 5′-Cy3-labelled 66mer DNA (Supplementary Table 1). smFRET experiments were performed as described above with 20 nM Cy5-labelled AID in a scanning buffer (50 mM MOPS, pH 7.4, 5 mM Mg^2+^, 60 mM Na^+^, 0.2 mg ml^−1^ BSA, 2 mM Trolox, 2.5 mM PCA and 250 nM PCD). Data were acquired at 100 ms time resolution for ∼5 min (ref. 59).

### smFRET for Cy5-AID scanning ssDNA

The ssDNA constructs were surface immobilized using a common anchor DNA (Supplementary Table 1) with 5′-Cy3 and 3′-biotin (Fig. 4). Excess DNA was washed out, and Cy5-labelled AID (20 nM) was introduced in the scanning buffer described above. Data were acquired at 30 ms or 1 s time resolution for ∼5 or ∼10 min, respectively. Low ionic strength experiments were performed similarly, but without Mg^2+^ and only 30 mM Na^+^ in all of the above buffers to reduce ionic strength. Single-step Cy5 photobleaching confirms that AID binds as a monomer under our experimental conditions.

### Post-synchronization analysis

To synchronize the long binding events with respect to their initial binding, we analyse the long binding FRET trajectories (Figs 4 and 5) from 10 s before the initial binding (threshold 0.15 FRET) until 10 s after the dissociation (0 FRET). Using a MATLAB script kindly provided by Jody Puglisi (Stanford University), we bin the trajectories (0.05 FRET bins, 2-s time bins) to determine how many trajectories are located at a given FRET and time value and generate the histograms. For the co-localization experiments (Fig. 3c), 0.1 FRET bins, five-frame time bins and threshold of 0.18 FRET were used. For the transcription bubble experiments (Fig. 3f), 0.05 FRET bins, 10 frame time bins and threshold of 0.35 FRET were used. This threshold value was chosen to avoid false starts owing to possibly noisy signals (<0.2 FRET). It was determined empirically to be optimal in order to properly synchronize all trajectories while staying clear of all signal fluctuations due to noise.

### FRET-ssDNA distance calibration experiments

We correlate FRET values to estimated substrate position using an empirical calibration with doubly labelled ssDNA with dye separations of 7, 15, 30, 45, 60, 60 and 72 nt between Cy3 and Cy5 (Supplementary Figs 17 and 18). ssDNA were surface-immobilized as described above, and data were acquired in the absence and presence of 20 nM AID at 15 ms or 1 s time resolution. The smFRET histograms were obtained by time-binning ∼100 background-corrected FRET trajectories. We collected a histogram of FRET values for each dye separation (Supplementary Fig. 17) and correlated the mean value with the distance between the dyes *q* (Supplementary Fig. 18b). Next, we used the mean FRET value for each dye separation distance to calculate donor–acceptor distance *R* using Förster's equation (Supplementary Fig. 18c), and fit the distance *R* to a linear combination of 
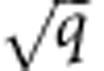
 (if substrate is a random coil) and *q* (if substrate is an extended chain) without bias. This fit yields 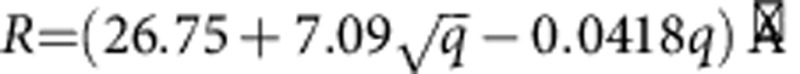 (Supplementary Fig. 18c, red line), which we used to convert FRET values directly to sequence positions *q*. Although this correlation allows us to map FRET to sequence position *q*, this mapping is only correct in an average sense, as there is a distribution of FRET values which correspond to the same *q* (Supplementary Fig. 17). However, this approximation is clearly valid at long distances, in which AID scans distances larger than the standard deviation of this distribution (see histograms in Supplementary Fig. 17). This calibration does not account for the size of AID, nor does it account for potential photophysical/rotational effects of the protein on the dyes. Therefore, it is not intended to yield an exact protein location, but rather an approximate nucleotide position, for which we use the term ‘estimated nucleotide position.'

### Mean square displacement analysis

Mean square displacement of Cy5-AID along ssDNA was obtained from long binding (*t*≥100 s) FRET trajectories for the hot motif, the product motif and pdT^41^. FRET trajectories (examples shown in Supplementary Fig. 20a,b) were used to estimate the nucleotide position of AID along the substrate using an experimental distance calibration (Supplementary Fig. 20c). The MSD was computed for each trajectory as a function of time (Supplementary Fig. 20d), the slope of which yields individual diffusion coefficients. The diffusion coefficients distribution histograms for each construct are reported in Fig. 6a–c.

## Additional information

**How to cite this article:** Senavirathne, G. *et al.* Activation-induced deoxycytidine deaminase (AID) co-transcriptional scanning at single-molecule resolution. *Nat. Commun.* 6:10209 doi: 10.1038/ncomms10209 (2015).

## Supplementary Material

Supplementary Information Supplementary Figures 1-22, Supplementary Tables 1-3 and Supplementary Reference
