# Supplementary material for: Activation-induced deoxycytidine deaminase (AID) co-transcriptional scanning at single-molecule resolution
Source: Nat Commun. 2015 Dec 18;6:10209. doi: 10.1038/ncomms10209 (PMC4703863; doi:10.1038/ncomms10209)
Supplement: Supplementary Information — Supplementary Figures 1-22, Supplementary Tables 1-3 and Supplementary Reference [file ncomms10209-s1.pdf]

Supplementary Information

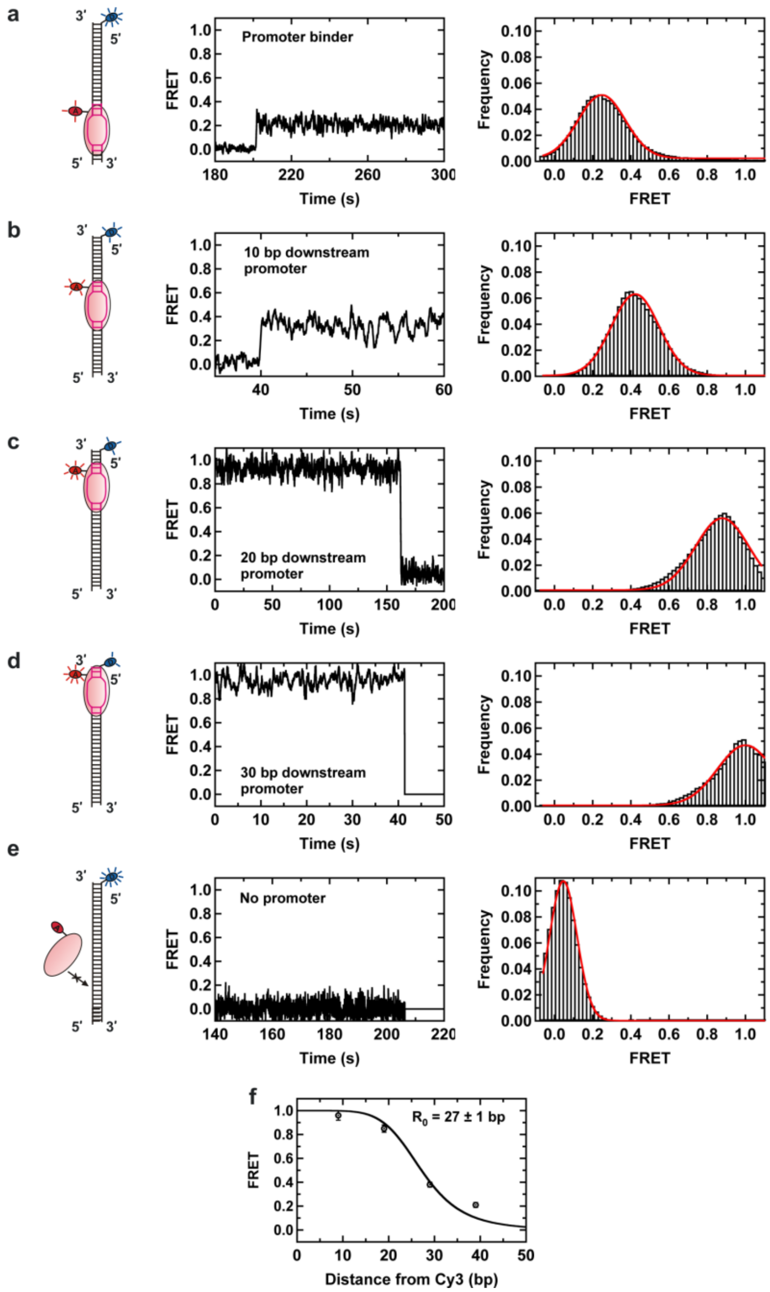

Supplementary Figure 1. Empirical calibration of Cy5-RNAP located with different promoter locations.

Single-molecule FRET diagram for visualizing Cy5-RNAP (FRET acceptor in red) on surface-immobilized Cy3-labeled dsDNA (FRET donor in blue) with different locations for the RNAP promoter (left panels). Representative FRET trajectories and histograms shown in the middle and right panels, respectively. (**a**) RNAP binds the promoter near the surface in the absence of NTPs at ~0.2 FRET (N = 71). This class of trajectories is identical in the presence of NTPs (Fig. 1b). Moving the promoter 10 (**b**), 20 (**c**), and 30 (**d**) base pairs downstream results in a FRET increase from to ~0.4 (N = 68), ~0.85 (N = 51) and ~0.95 (N = 24), respectively. (**e**) Cy5-RNAP does not bind dsDNA in the absence of a promoter and NTPs (zero FRET observed, N = 51). (**f**) Empirical FRET calibration of the distance between the Cy3 on the distal end of the dsDNA and Cy5-RNAP at each promoter location. A fit to the Förster's equation yields apparent R_0 = 27 ± 1 bp (standard error of the fit). The time resolution of the PB event shown in (a) is 100 ms, while the histogram contains events obtained at 15 ms resolution. All remaining events were obtained at 30 ms (b-d) or 15 ms (e) time resolution.

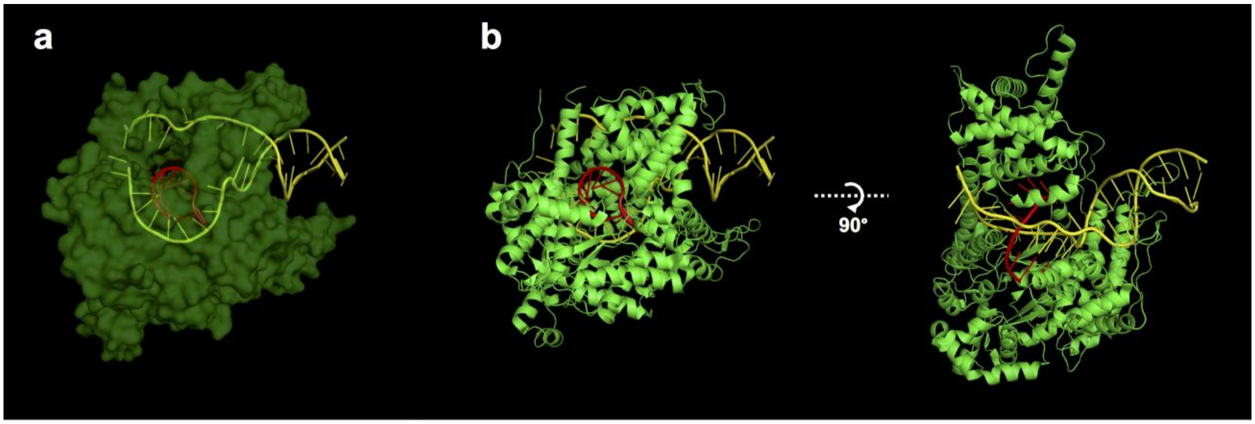

Supplementary Figure 2. Bound RNAP distorts the DNA.

Crystal structure of T7 RNAP (green, PDBID 1MSW) bound to DNA (yellow) and nascent RNA (red). **(a)** The structure shows a large ssDNA region in the active site, and (**b**) the downstream dsDNA bent towards the N-terminus of the RNAP.

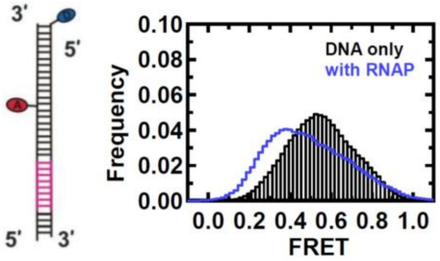

Supplementary Figure 3. RNAP binding distorts dsDNA structure.

Surface-immobilized dsDNA was labeled with Cy3 on the distal end (FRET donor in blue), and Cy5 10 bp downstream from the promoter (FRET acceptor in red). FRET distribution shifts from ~0.5 in the absence of protein (black, N = 61) to ~0.3 (blue, N = 62) in the presence of RNAP, consistent with RNAP distorting the dsDNA structure around the promoter binding site. Experimental time resolution is 30 ms.

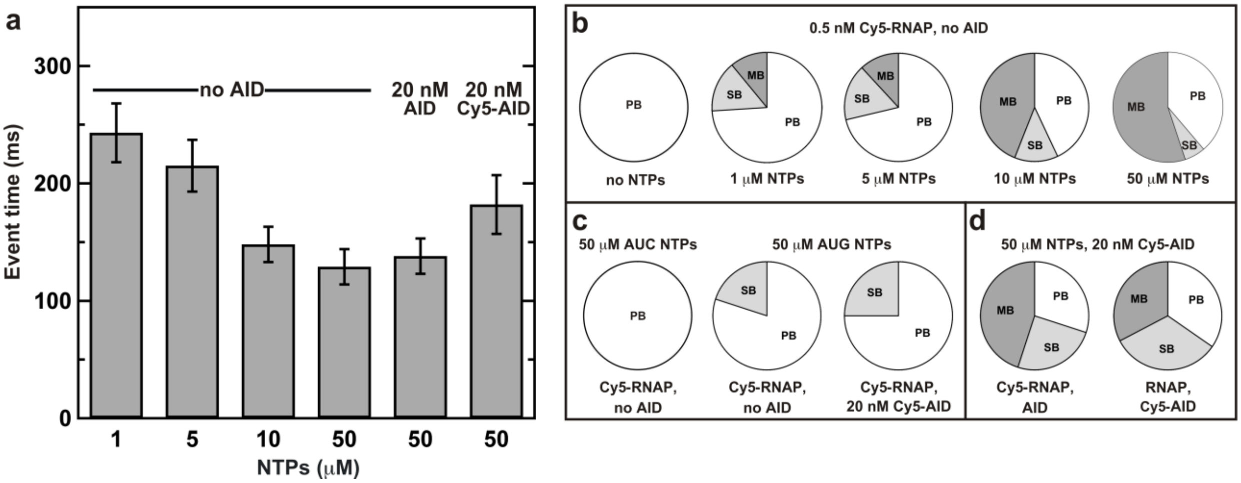

Supplementary Figure 4. RNAP trajectories depend upon NTP concentration.

(a) The average time of an RNAP MB decreases with increasing NTP concentration. The average MB time increases in the presence of unlabeled AID (N = 24) and Cy5-labeled AID (N = 20), indicating that AID slows down active RNAP. Error bars reflect standard error of the mean (SEM) unless it was less than the time resolution of the experiment, in which cases the time resolution was used as the uncertainty in the measurement. This effect does not depend on whether RNAP or AID is labeled. (**b**) Fraction of moving bubbles increases with NTP concentration (SEM, N >50, Supplementary Table 2). (**c**) In the absence of GTP, RNAP does not leave the promoter. In the absence of CTP, RNAP stalls at the single G (0.4 FRET), which is also observed in the presence of Cy5-AID. (**d**) In the presence of 20 nM AID, stalled bubbles (N = 53) increase dramatically (compare to right panel of (**b**) above, 50 µM NTPs), suggesting that AID stalls active transcription. All events used for this analysis were obtained at 15 or 30 ms time resolution.

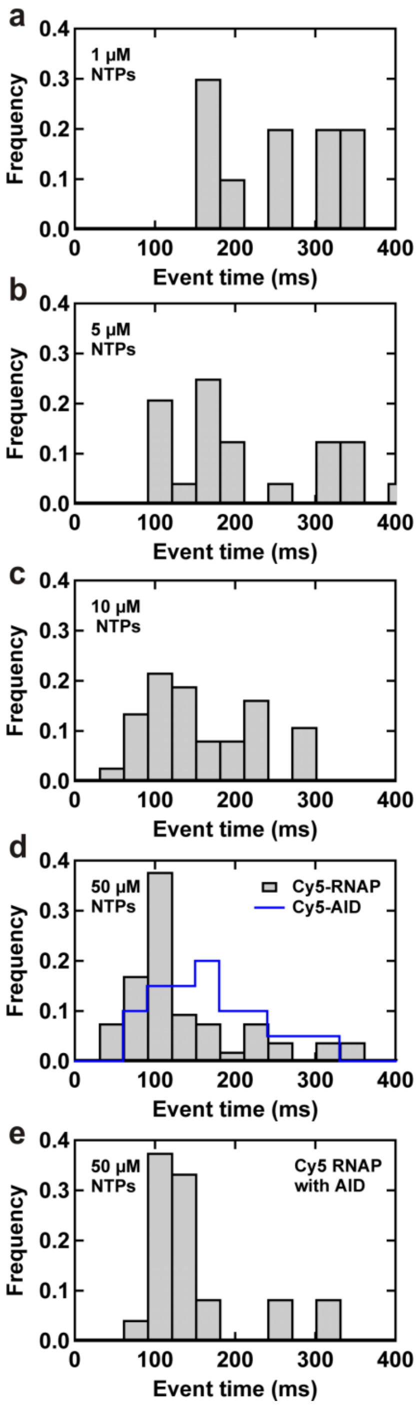
a
1 μM NTPs
b
5 μM NTPs
c
10 μM NTPs
d
50 μM NTPs
Cy5-RNAP
Cy5-AID
e
50 μM NTPs
Cy5 RNAP with AID
Frequency
Event time (ms)

Supplementary Figure 5. Distributions of RNAP transcription time depend upon NTP concentration.

Distribution of MB peak rise times τ for Cy5-RNAP in the presence of (**a**) 1 μM (N = 10), (**b**) 5 μM (N = 24), (**c**) 10 μM (N = 37), and (**d**) 50 μM NTPs (N = 53, gray bars). The distribution shifts towards longer times in the presence of Cy5-AID (N = 20, compare blue line to gray bars). (**e**) Distribution of peak rise times for Cy5-RNAP in the presence of AID and 50 μM NTPs (N = 24). There are few events at low NTP concentrations due to limited number of MB events observed (see Supplementary Fig. 4). All events used for this analysis were obtained at 15 or 30 ms time resolution.

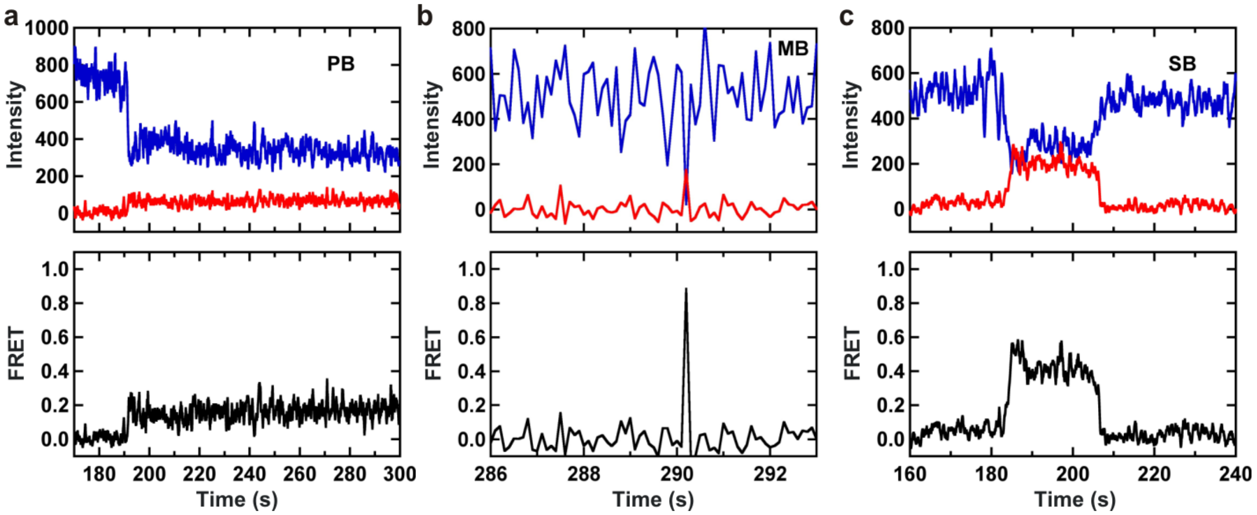

Supplementary Figure 6. Characteristic single molecule fluorescence and FRET trajectories with Cy5-RNAP during transcription.

Representative fluorescence and FRET trajectories of (**a**) PB, (**b**) MB, and (**c**) SB events in the presence of NTPs. The time resolution of FRET trajectories shown here is 100 ms, and these experiments were also conducted at 15-30 ms time resolution (Supplementary Figs 4-9).

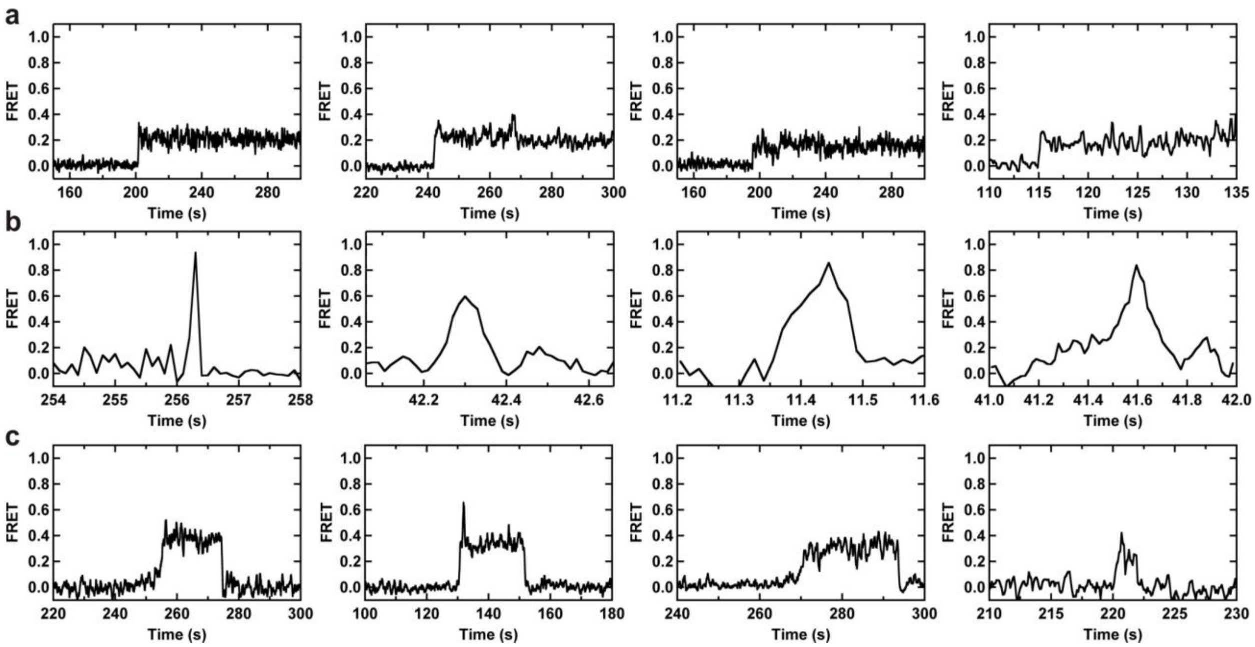

Supplementary Figure 7. Single molecule FRET trajectories of Cy5-RNAP transcription of dsDNA.

Single-molecule FRET trajectories of (**a**) PB, (**b**) MB, and (**c**) SB events for Cy5-RNAP on Cy3-labeled dsDNA. The time resolution of FRET trajectories shown is 100 ms (a, b panel 1, and c) or 15 ms (b, panels 2-4).

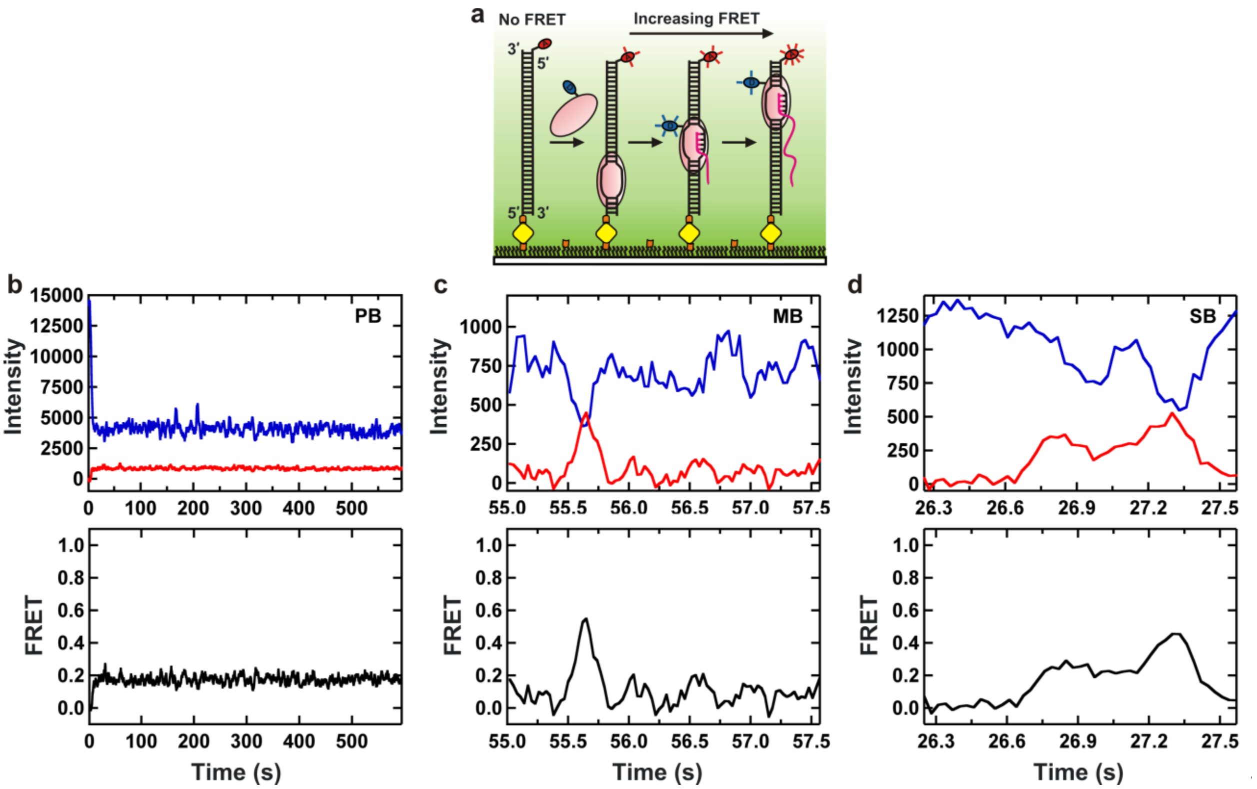

Supplementary Figure 8. Single molecule fluorescence and FRET trajectories with Cy3-RNAP.

(**a**) Single-molecule FRET setup for visualizing Cy3-RNAP (FRET donor in blue) on surface-immobilized Cy5-labeled dsDNA (FRET acceptor in red) with an RNAP promoter near the surface. (**b**) Representative single molecule intensity and FRET trajectories of a PB event in the absence of NTPs. This class of trajectories is identical in the presence of NTPs. Representative trajectories of (**c**) MB and (**d**) SB events in the presence of NTPs. The time resolution of FRET trajectories shown here is 30 ms.

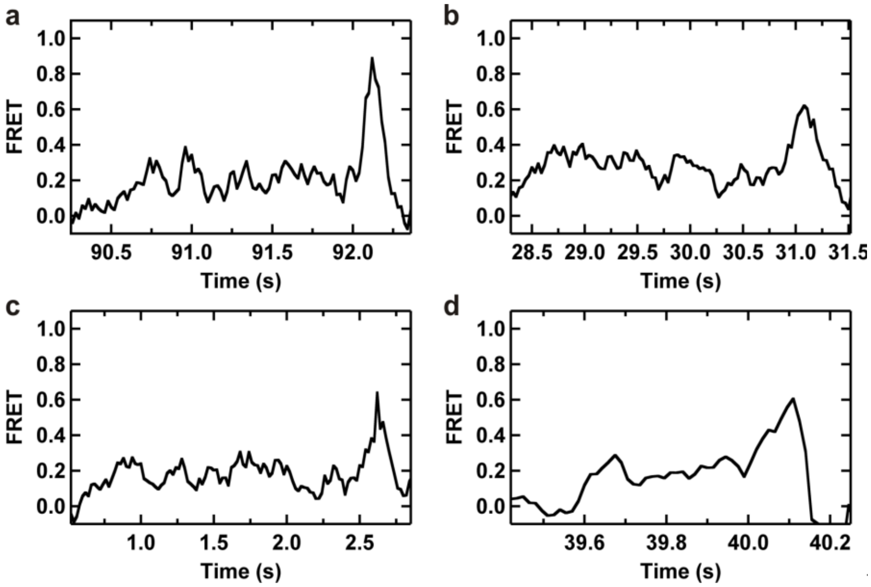

Supplementary Figure 9. RNAP first binds the promoter, followed by translocation.

(**a-d**) Single-molecule FRET trajectories in which RNAP first binds the promoter at 0.2 FRET for up to ~2 seconds, followed by translocation towards the distal end (high FRET) and then dissociation in the presence of NTPs. These trajectories are not observed in the absence of NTPs. The time resolution of FRET trajectories shown here is 20 ms (a and c), 30 ms (b), or 15 ms (d).

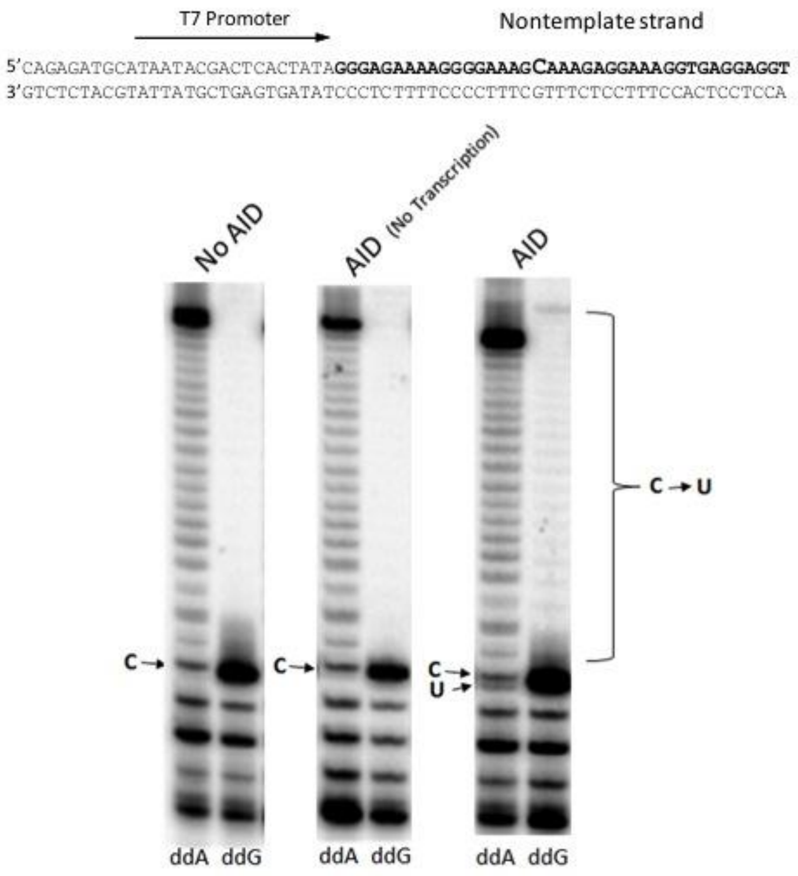

Supplementary Figure 10. Transcription-dependent deamination of AID on dsDNA.

Sequence of dsDNA substrate used in smFRET experiments. The non-template strand contains a single dC used to measure deamination by AID (top). Polyacrylamide gel electrophoresis (PAGE) data showing AID deamination of dC to dU on the non-transcribed strand of dsDNA is dependent on transcription (lanes 5 and 6), as previously reported[1]. dC → dU is measured by the presence of a termination band when ddA is used instead of dA (U →, lane 5 ddA), as well as by the presence of bands migrating past the dC → dU template site, when ddG is used instead of dG, denoted by the bracket to the right of the gel (ddG, lane 6) (bottom).

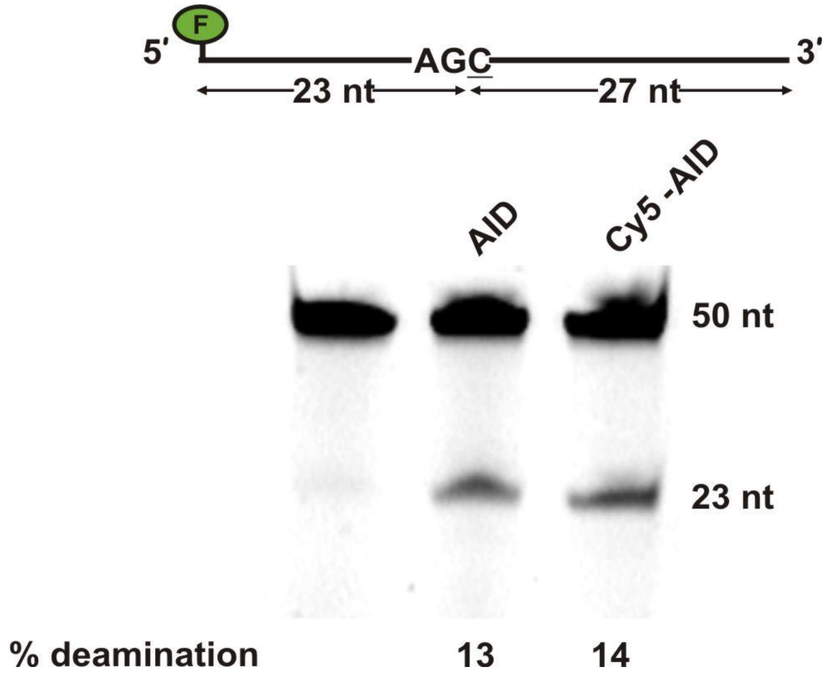

Supplementary Figure 11. Cy5-labeled and unlabeled AID have similar activities. AID or Cy5-AID activity was measured on a 50 nt ssDNA substrate containing a single AGC motif (top). Following dC to dU conversion, the DNA was incubated with uracil glycosylase to generate an abasic site at the dU, and then alkali treated to cleave the 50 nt substrate at the abasic site. Deamination was detected by separating the fluorescein-labeled DNA fragment (23 nt) from full-length substrate (50 nt) using PAGE. Gel band intensities were used to quantify percent deamination (bottom).

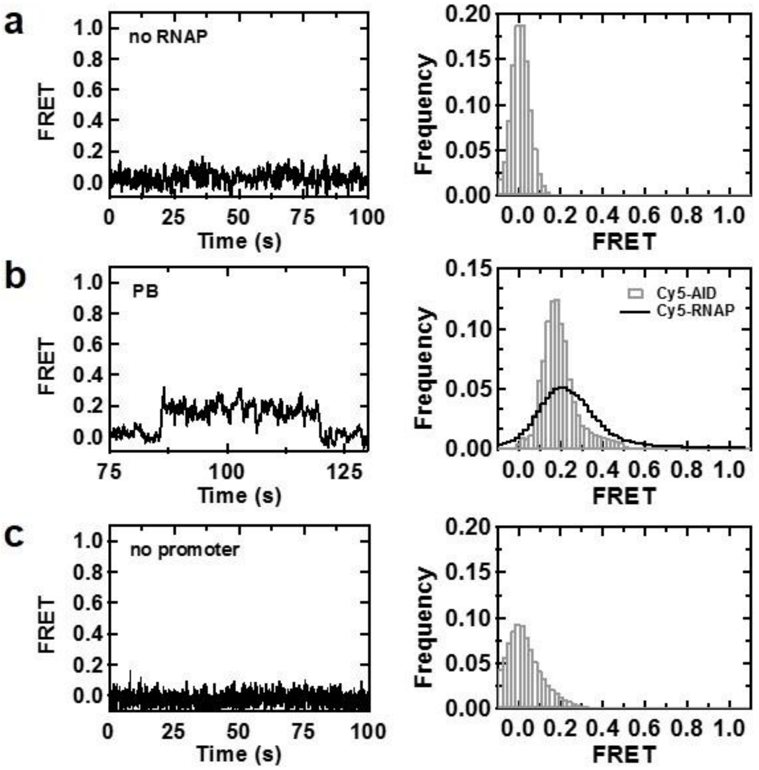

Supplementary Figure 12. Single molecule FRET trajectories and histograms with Cy5-AID.

(**a**) Cy5-AID does not bind dsDNA in the absence of RNAP (0 FRET observed). (**b**) Representative smFRET trajectory of a promoter binding (PB) event in the absence of NTPs. This class of trajectories is identical in the presence of NTPs (compare to Fig. 2b). FRET histograms for Cy5-AID (gray, 100 ms time resolution) and Cy5-RNAP in the absence of AID (black, 15 ms time resolution) are both centered at 0.2 FRET. (**c**) Cy5-AID does not bind a dsDNA lacking a T7 promoter even in the presence of RNAP (0 FRET observed). The time resolution of FRET trajectories shown here is 100 ms (a and b) and 15 ms (c). FRET histogram shown in panel (c) is broader than that shown in panel (a) due to the increase in time resolution.

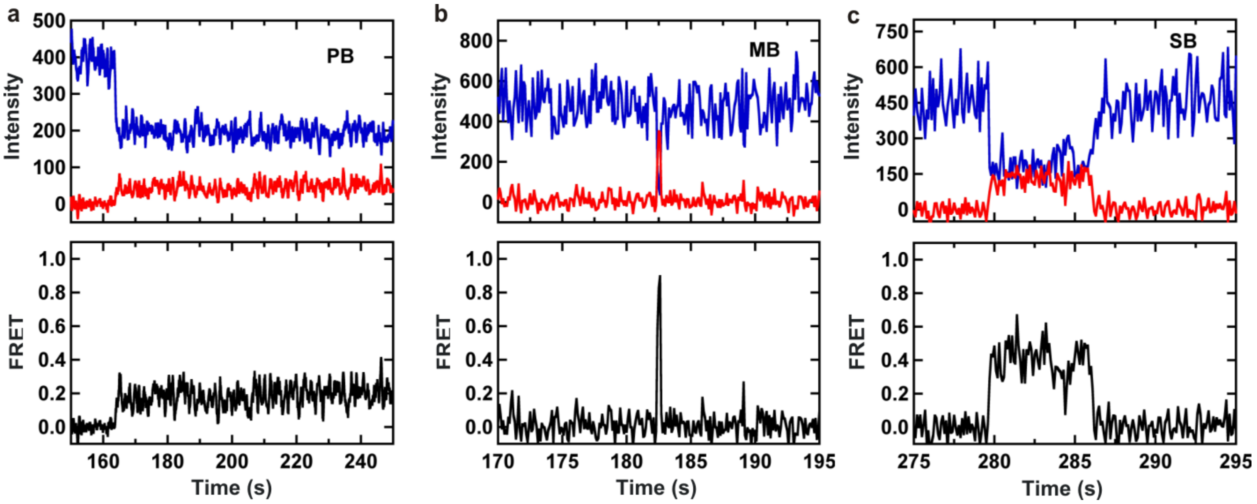

Supplementary Figure 13. Characteristic single molecule fluorescence and FRET trajectories with Cy5-AID during transcription.

Representative fluorescence and FRET trajectories of (**a**) PB, (**b**) MB, and (**c**) SB events in the presence of NTPs. The time resolution of FRET trajectories shown here is 100 ms and these experiments were also conducted at 15-30 ms (Supplementary Figs 4-5 and 12-13).

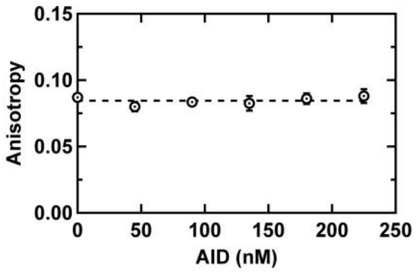

Supplementary Figure 14. T7 RNAP and AID do not interact directly.

The rotational anisotropy of fluorescein-labeled RNAP (50 nM) was measured at increasing concentrations of AID. No change in anisotropy was observed indicating the lack of direct interaction between AID and RNAP.

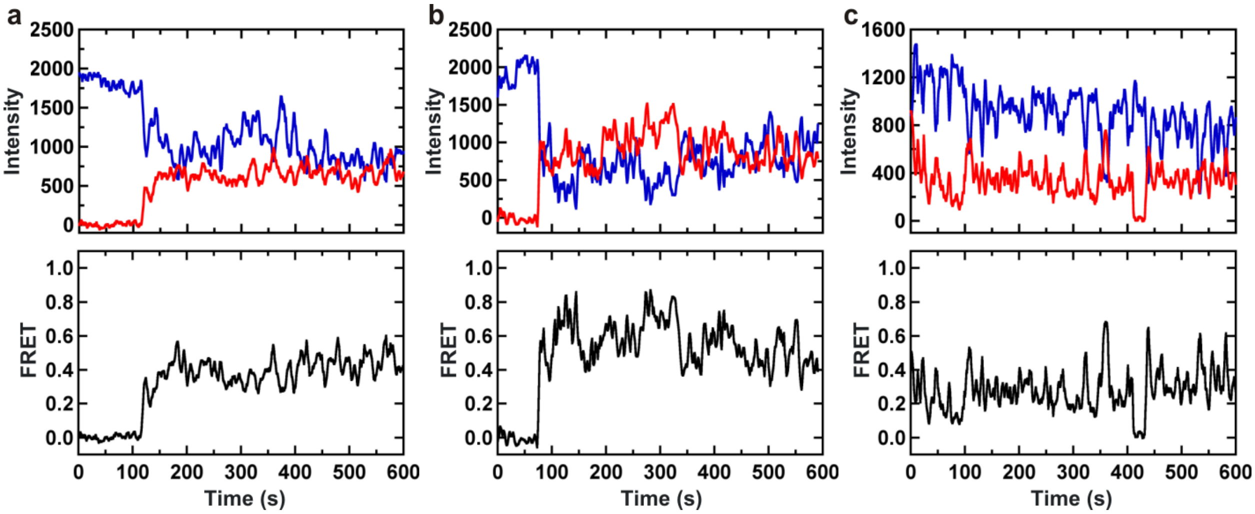

Supplementary Figure 15. Single molecule trajectories for AID bound to pdT 5′ hot for almost 10 min.

Representative fluorescence and smFRET long time trajectories for 5′ AAC hot; (top) donor (blue) and acceptor (red) intensities, (bottom) corresponding FRET ratios over a given time. The time resolution of FRET trajectories in these experiments is 1 s.

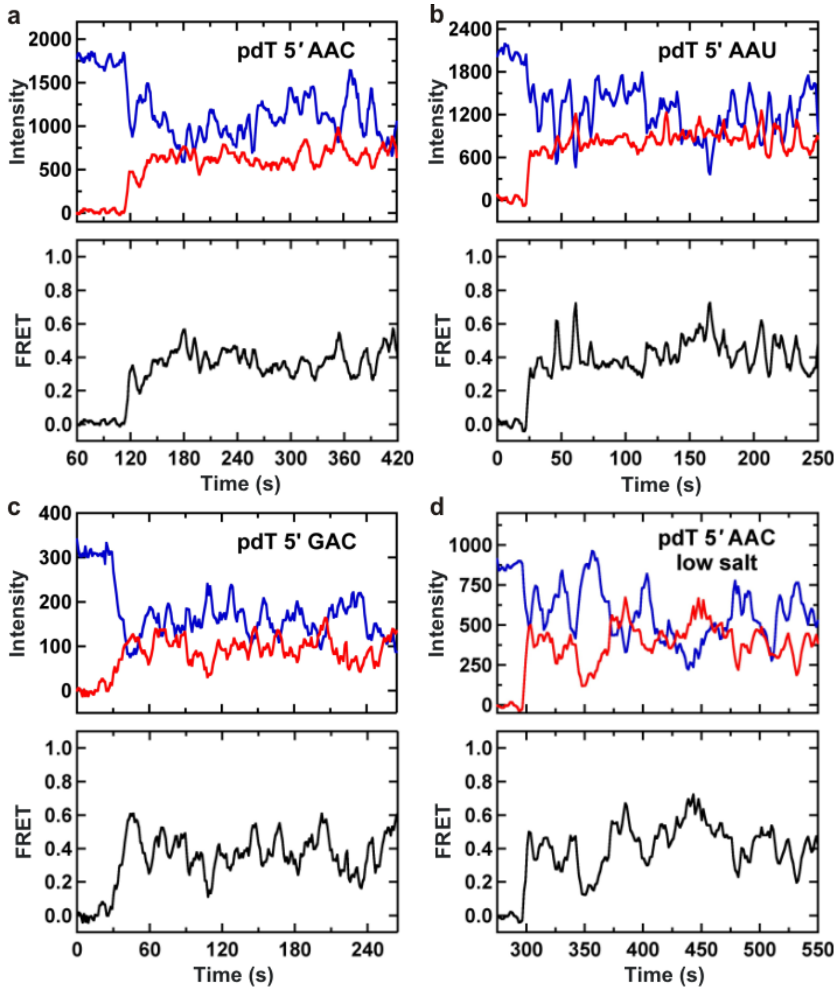

Supplementary Figure 16. Single molecule intensity trajectories and corresponding smFRET trajectories for scanning of AID on different pdT substrates.

Representative single molecule fluorescence and FRET time trajectories; donor (blue) and acceptor (red) intensities with corresponding calculated smFRET ratios below, as indicated for each substrate. The time resolution of these experiments is 1 s.

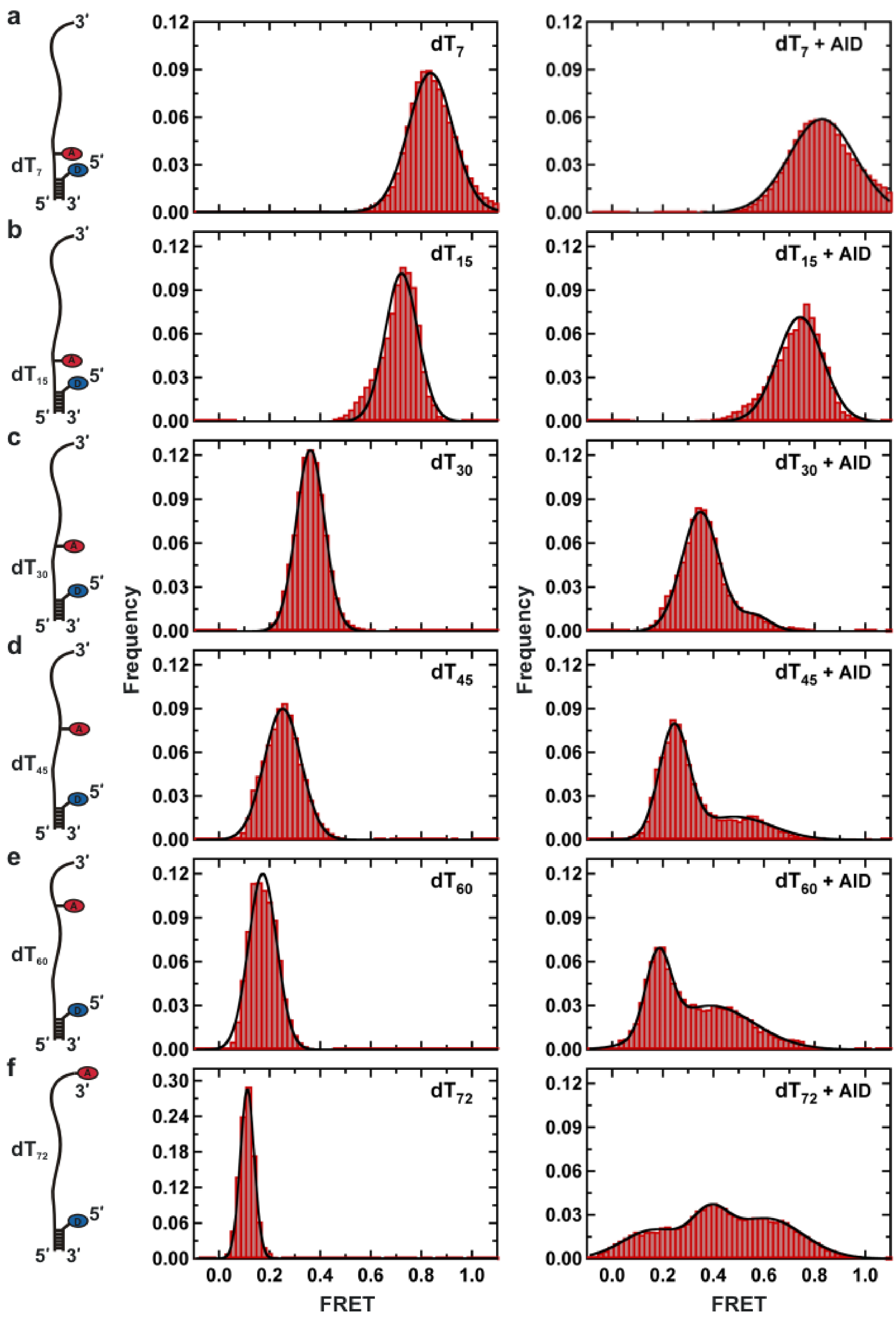
a
b
c
d
e
f
dT7
dT15
dT30
dT45
dT60
dT72
dT7 + AID
dT15 + AID
dT30 + AID
dT45 + AID
dT60 + AID
dT72 + AID
Frequency
FRET
3'
5'
A
D

Supplementary Figure 17. FRET histograms for ssDNA calibration constructs.

Cy3-Cy5-labeled ssDNA constructs used for the calibration experiments (panel 1, also shown in Supplementary Fig. 18a). The distance between Cy3 and Cy5 was increased as indicated. ~100 smFRET trajectories were combined to compile time averaged FRET distributions for each ssDNA construct, in the absence of (panel 2), and in the presence of (panel 3) unlabeled AID. The resulting average FRET value for each distribution (see Supplementary Fig. 18b) was converted into a distance using the Förster's equation, and plotted in Supplementary Fig. 18c as a function of Cy3-Cy5 separation. The time resolution of these experiments is 15 ms (a) or 1 s (b-f).

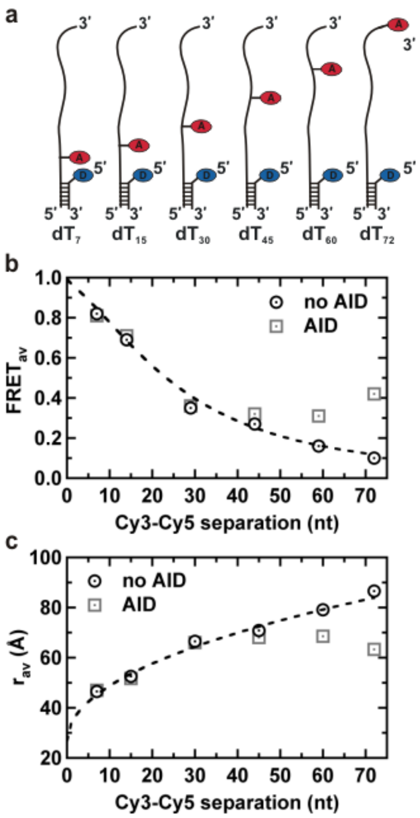

Supplementary Figure 18. FRET-ssDNA distance calibration.

(**a**) Doubly-labeled ssDNA constructs used for the calibration experiments, the separation between FRET pairs are indicated each for construct. (**b**) Average FRET values for each construct (see Supplementary Fig. 17) in the absence (black) and presence (gray) of AID, as a function of nucleotide separation. (**c**) Average distances between FRET pairs in Angstroms, calculated from the average FRET distributions (Supplementary Fig. 17) in the absence (black) and presence (gray) of AID, as a function of nucleotide separation. FRET and distance distributions in the absence of AID fit a Guassian model (dashed lines). The time resolution of these experiments is 1 s.

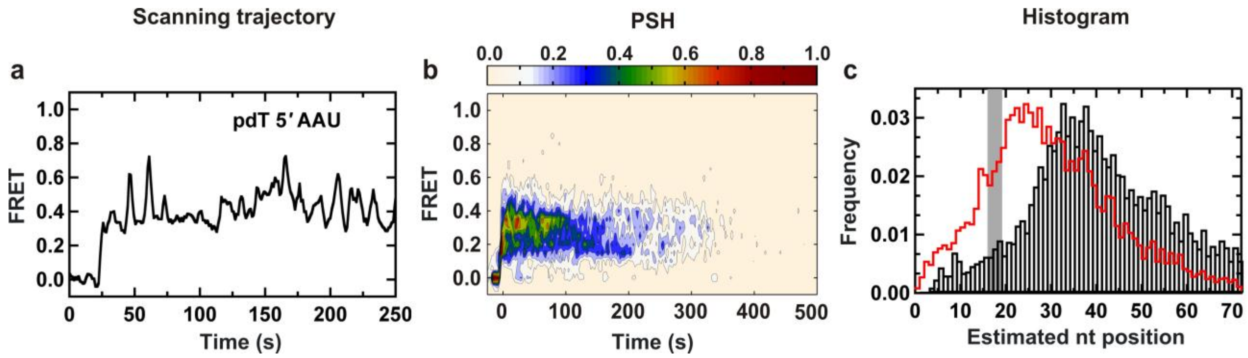

Supplementary Figure 19. AID scans the AAU product motif.

(**a**) Representative FRET trajectory for AID scanning of ssDNA with a single AAU product motif. (**b**) Replacing the AAC hot motif with the AAU product motif results in a shorter PSH tail, and AID scanning shifts towards the 3′ end (~0.3 FRET) relative to the AAC hot motif (~0.4 FRET, Fig. 4b). (**d**) The estimated sequence position histogram clearly indicates that AID scanning preference shifts away from the product motif (5′ AAC hot histogram shown in red for comparison). The time resolution of these experiments is 1 s.

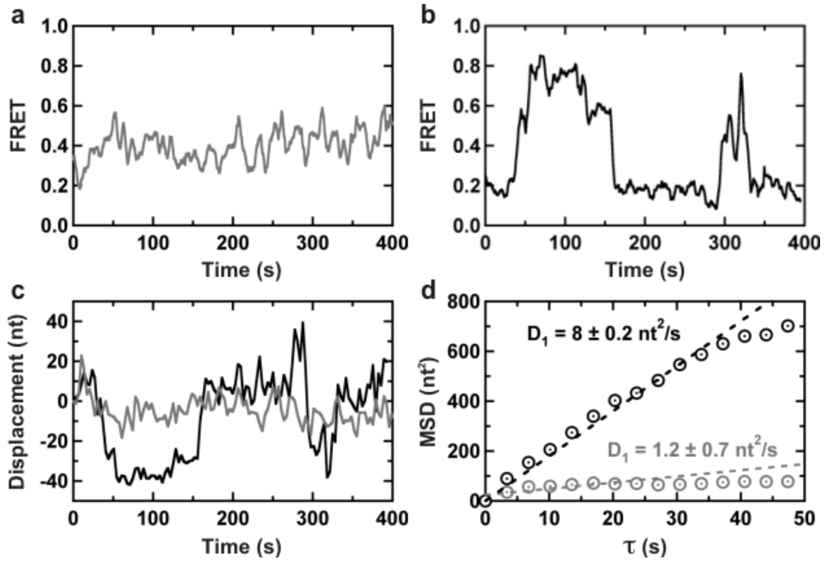

Supplementary Figure 20. Mean squared displacement analysis of AID on 5′ AAC pdT.

Representative FRET trajectories of AID (**a**) hovering over a narrow range of nucleotide positions, and (**b**) traversing the entire length of the substrate. (**c**) The corresponding AID displacement from the initial FRET value, for the narrow (gray) and long (black) trajectories, calculated from average FRET distributions (Supplementary Fig. 18b), as a function of time. (**d**) Mean square displacement as a function of time, where the fit to the initial linear region (dashed lines) yields D_1. The time resolution of these experiments is 1 s.

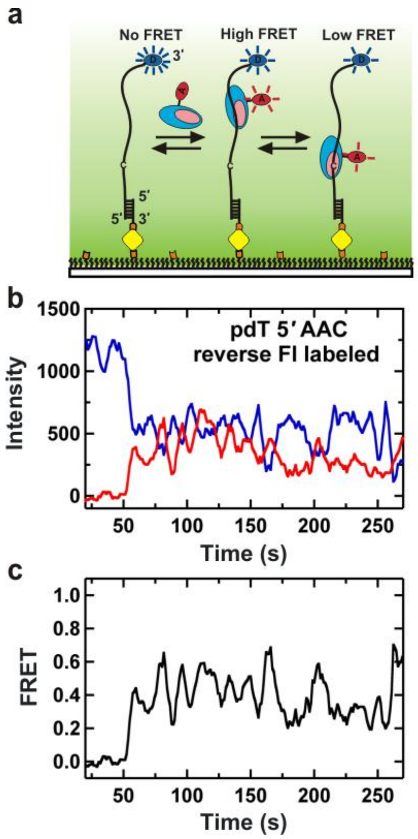

Supplementary Figure 21. AID scans ssDNA from the 3′ end.

(**a**) Single-molecule FRET setup for analyzing scanning of AID on ssDNA relative to the free 3′ end. 3′-Cy3-labeled ssDNA (92 nt) is annealed to a surface immobilized anchor DNA. Binding and scanning of Cy5-labeled (A = acceptor) AID results in FRET changes. (**b**) Representative fluorescence intensities and (**c**) FRET ratios as a function of time. The time resolution of these experiments is 1 s.

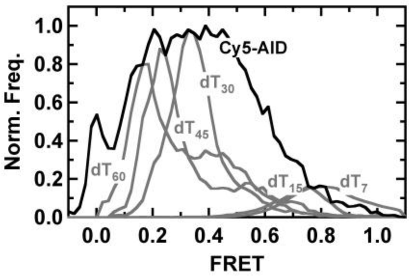

Supplementary Figure 22. Deconvolution of AID-ssDNA scanning and AID-induced ssDNA conformational dynamics.

FRET distribution for Cy5-AID scanning on 5′-Cy3 dT_{72} ssDNA is overlapped with the FRET distributions obtained from calibration experiments for doubly labeled ssDNA with (unlabeled) AID (shown in Supplementary Figs 17-18). Amplitude of the Cy5-AID histogram is normalized to 1, and amplitudes of the histograms for doubly labeled ssDNA are renormalized such that they are 'enveloped' by the Cy5-AID scanning histogram.

Name	Sequence (5′→3′)
5′-Biotin 69-mer DNA	Biotin-TTT CAG AGA TGC ATA ATA CGA CTC ACT ATA GGG AGA AAA GGG GAA **AGC** AAA GAG GAA AGG TGA GGA GGT
5′-Cy3 66-mer DNA[1]	Cy3-ACC TCC TCA CCT TTC CTC TTT GCT TTC CCC TTT TCT CCC TAT AGT GAG TCG TAT TAT GCA TCT CTG
5′-Cy3 8 nt mismatch 66-mer DNA[1]	Cy3-ACC TCC TCA CCT TTC CTC AAA AAA AAC CCC TTT TCT CCC TAT AGT GAG TCG TAT TAT GCA TCT CTG
5′-Biotin 66-mer No promoter DNA	Biotin-CAG AGA TGC AGC CGT CCG TAT CCG TAT TGT AGA AAA GGG GAA AGC AAA GAG GAA AGG TGA GGA GGT
5′-Cy3 66-mer No promoter DNA[2]	Cy3-ACC TCC TCA CCT TTC CTC TTT GCT TTC CCC TTT TCT ACA ATA CGG ATA CGG ACG GCT GCA TCT CTG
5′-Biotin 69-mer 10 bp downstream promoter DNA	Biotin-TTT GTG AGG AGG TCA GAG ATG CAT AAT ACG ACT CAC TAT AGG GAG AAA AGG GGA AAG CAA AGA GGA AAG
5′-Cy3 66-mer 10 bp downstream promoter DNA[3]	Cy3-CTT TCC TCT TTG CTT TCC CCT TTT CTC CCT ATA GTG AGT CGT ATT ATG CAT CTC TGA CCT CCT CAC
5′-Biotin 69-mer 20 bp downstream promoter DNA	Biotin-TTT AAG AGG AAA GGT GAG GAG GTC AGA GAT GCA TAA TAC GAC TCA CTA TAG GGA GAA AAG GGG AAA GCA
5′-Cy3 66-mer	Cy3-TGC TTT CCC CTT TTC TCC CTA TAG TGA GTC GTA TTA

20 bp downstream promoter DNA[4]	TGC ATC TCT GAC CTC CTC ACC TTT CCT CTT
5′-Biotin 69-mer 30 bp downstream promoter DNA	Biotin-TTT GGG GAA AGC AAA GAG GAA AGG TGA GGA GGT CAG AGA TGC ATA ATA CGA CTC ACT ATA GGG AGA AAA
5′-Cy3 66-mer 30 bp downstream promoter DNA[5]	Cy3-TTT TCT CCC TAT AGT GAG TCG TAT TAT GCA TCT CTG ACC TCC TCA CCT TTC CTC TTT GCT TTC CCC
5′-Biotin 69-mer promoter +10 bp Cy5-dT DNA	Biotin-TTT CAG AGA TGC ATA ATA CGA CTC ACT ATA GGG AGA AAA G(Cy5-T)G GAA AGC AAA GAG GAA AGG TGA GGA GGT
5′-Cy3 66-mer promoter +10 bp DNA[6]	Cy3-ACC TCC TCA CCT TTC CTC TTT GCT TTC CAC TTT TCT CCC TAT AGT GAG TCG TAT TAT GCA TCT CTG
Anchor DNA	Cy3-CGC GAG GAA TGG ATG TAG GG-Biotin
pdT 5′ AAC[7]	CCC TAC ATC CAT TCC TCG CG TTT TTT TTT TTT TTT **AAC** TTT TTT TTT TTT TTT TTT TTT TTT TTT TTT TTT TTT TTT TTT TTT TTT TTT TTT
pdT 5′ AAU[7]	CCC TAC ATC CAT TCC TCG CG TTT TTT TTT TTT TTT **AAU** TTT TTT TTT TTT TTT TTT TTT TTT TTT TTT TTT TTT TTT TTT TTT TTT TTT TTT
pdT 5′ GAC[7]	CCC TAC ATC CAT TCC TCG CG TTT TTT TTT TTT TTT **GAC**

	TTT TTT TTT TTT TTT TTT TTT TTT TTT TTT TTT TTT TTT TTT TTT TTT TTT TTT
pdT[7]	CCC TAC ATC CAT TCC TCG CG TTT TTT TTT TTT TTT TTT TTT TTT TTT TTT TTT TTT TTT TTT TTT TTT TTT TTT TTT TTT TTT TTT TTT TTT
pdT 5′ $(WRC)_3$– $(GAC)_3$ 3′ [7]	CCC TAC ATC CAT TCC TCG CG TTT TTT TTT TTT TTT **AAC AGC AAC** TTT TTT TTT TTT TTT TTT TTT TTT TTTT **GAC GAC GAC** TTT TTT TTT TT
pdT 5′ $(GAC)_3$– $(WRC)_3$ 3′ [7]	CCC TAC ATC CAT TCC TCG CG TTT TTT TTT TTT TTT **GAC GAC GAC** TTT TTT TTT TTT TTT TTT TTT TTT TTTT **AAC AGC AAC** TTT TTT TTT TT
pdT 5′ $(WRU)_3$– $(GAU)_3$ 3′ [7]	CCC TAC ATC CAT TCC TCG CG TTT TTT TTT TTT TTT **AAU AGU AAU** TTT TTT TTT TTT TTT TTT TTT TTT TTTT **GAU GAU GAU** TTT TTT TTT TT

Supplementary Table 1. DNA sequences used in this study.

AID targets are highlighted in bold. T7 promoter sequences are highlighted in red, nucleotides changed to remove T7 promoter are highlighted in green, and 8 pdA to form 8 nt mismatch are highlighted in blue.

[1]Complementary to 5′-Biotin 69-mer DNA

[2]Complementary to 5′-Biotin 66-mer No promoter DNA

[3]Complementary to 5′-Biotin 69-mer 10 bp downstream promoter DNA

[4]Complementary to 5′-Biotin 69-mer 20 bp downstream promoter DNA

[5]Complementary to 5′-Biotin 69-mer 30 bp downstream promoter DNA

[6]Complementary to 5′-Biotin 69-mer promoter +10 bp Cy5-dT DNA

[7]Complementary to Anchor DNA

[T7 RNAp] (nM)	[NTP] (μM)	[AID] (nM)	Cy5 Label	N
0.5	0	0	RNAP	71
0.5	1	0	RNAP	85
0.5	5	0	RNAP	78
0.5	10	0	RNAP	87
0.5	50	0	RNAP	84
0.5	50	20	RNAP	53
0.5	50	20	AID	55
0	0	20	AID	112
0.5	0	20	AID	108
3 NTPs only				
0.5	50 (A,U,C)	0	RNAP	246
0.5	50 (A,U,G)	0	RNAP	131
0.5	50 (A,U,G)	20	AID	116
No Promoter DNA				
0.5	0	0	RNAP	51
0.5	0	20	AID	245
Downstream Promoter DNA				
0.5	0	0	RNAP	51

Supplementary Table 2. Total number of trajectories observed for each RNAP experiment.

Total number of trajectories *N* observed for each of the experimental conditions, in the presence and absence of RNAP, NTPs, and AID, on dsDNA constructs with different locations for the T7 RNAP promoter.

DNA	Number of Molecules (N)	Number of Short Binders	Number of Long Binders	Average bound time (s)
pdT 5′ AAC	118	17	101	273 ± 27
pdT 5′ AAU	102	20	82	217 ± 24
pdT 5′ GAC	93	18	75	213 ± 25
pdT 5′ AAC low salt	96	11	85	290 ± 30
pdT	111	21	90	216 ± 23
pdT 5′ $(WRC)_3$–$(GAC)_3$ 3′	72	18	54	222 ± 30
pdT 5′ $(GAC)_3$–$(WRC)_3$ 3′	48	7	41	202 ± 30
pdT 5′ $(WRU)_3$–$(GAU)_3$ 3′	67	13	54	192 ± 24

Supplementary Table 3. Fraction of short and long binders for each AID substrate and average bound times for long binders.

Total number of binding events *N* classified into short and long binding events, with average bound times (SEM) for long binding events.

References

1 Pham, P., Bransteitter, R., Petruska, J. & Goodman, M. F. Processive AID-catalysed cytosine deamination on single-stranded DNA simulates somatic hypermutation. *Nature* **424**, 103-107, doi:10.1038/nature01760 (2003).
